# Atypical enteropathogenic *E. coli* are associated with disease activity in ulcerative colitis

**DOI:** 10.1080/19490976.2022.2143218

**Published:** 2022-11-22

**Authors:** Maximilian Baumgartner, Rebecca Zirnbauer, Sabine Schlager, Daniel Mertens, Nikolaus Gasche, Barbara Sladek, Craig Herbold, Olga Bochkareva, Vera Emelianenko, Harald Vogelsang, Michaela Lang, Anton Klotz, Birgit Moik, Athanasios Makristathis, David Berry, Stefanie Dabsch, Vineeta Khare, Christoph Gasche

**Affiliations:** a Division of Gastroenterology and Hepatology, Department of Internal Medicine 3, Medical University of Vienna, Vienna, Austria; b Division of Visceral Surgery, Department of General Surgery, Medical University of Vienna, Vienna, Austria; c National Reference Laboratory for Escherichia coli, Austrian Agency for Health and Food Safety, Graz, Austria; d Biome Diagnostics GmbH, Vienna, Austria; e Joint Microbiome Facility of the Medical University of Vienna and the University of Vienna, Vienna, Austria; f Institute of Science and Technology Austria, Klosterneuburg, Austria; g Centre for Microbiology and Environmental Systems Science, Department of Microbiology and Ecosystem Science, Division of Microbial Ecology, University of Vienna, Vienna, Austria; h Division of Microbiology, Department of Laboratory Medicine, Medical University of Vienna, Vienna, Austria

**Keywords:** Ulcerative colitis (UC), Crohn's disease (CD), inflammatory bowel disease (IBD), 5-ASA, p21-activated kinase (PAK), enteropathogenic E. coli (EPEC), Escherichia coli (E. coli), effector proteins, virulence factors, bacterial–epithelial interaction, microbiome

## Abstract

With increasing urbanization and industrialization, the prevalence of inflammatory bowel diseases (IBDs) has steadily been rising over the past two decades. IBD involves flares of gastrointestinal (GI) inflammation accompanied by microbiota perturbations. However, microbial mechanisms that trigger such flares remain elusive. Here, we analyzed the association of the emerging pathogen atypical enteropathogenic *E. coli* (aEPEC) with IBD disease activity. The presence of diarrheagenic *E. coli* was assessed in stool samples from 630 IBD patients and 234 age- and sex-matched controls without GI symptoms. Microbiota was analyzed with 16S ribosomal RNA gene amplicon sequencing, and 57 clinical aEPEC isolates were subjected to whole-genome sequencing and in vitro pathogenicity experiments including biofilm formation, epithelial barrier function and the ability to induce pro-inflammatory signaling. The presence of aEPEC correlated with laboratory, clinical and endoscopic disease activity in ulcerative colitis (UC), as well as microbiota dysbiosis. In vitro, aEPEC strains induce epithelial p21-activated kinases, disrupt the epithelial barrier and display potent biofilm formation. The effector proteins *espV* and *espG2* distinguish aEPEC cultured from UC and Crohn’s disease patients, respectively. EspV-positive aEPEC harbor more virulence factors and have a higher pro-inflammatory potential, which is counteracted by 5-ASA. aEPEC may tip a fragile immune–microbiota homeostasis and thereby contribute to flares in UC. aEPEC isolates from UC patients display properties to disrupt the epithelial barrier and to induce pro-inflammatory signaling in vitro.

## Introduction

Ulcerative colitis (UC) and Crohn’s disease (CD) are the most prevalent forms of inflammatory bowel diseases (IBD) and affect 0.5 − 1% of the Western population. IBD is characterized by chronically remittent inflammation of the gastrointestinal tract, associated with abdominal pain, diarrhea, intestinal mucosal ulceration and anemia. The molecular pathogenesis of IBD involves a complex interplay of host genetics, aberrant mucosal immune response, epithelial barrier dysfunction, gut microbiome and environmental triggers that interfere with such. Genome-wide association studies have identified more than 240 risk loci which have been linked to an aberrant immune–microbiota homeostasis and intestinal barrier dysfunction.^1,2^ IBD prevalence increases in an industrialized environment, more specific with the use of food additives and pharmaceuticals that interfere with microbiota or barrier function. Emulsifiers, titanium dioxide, ethylenediaminetetraacetate acid (EDTA), NSAR and antibiotics serve as examples.^3–6^

The microbiota of IBD patients is characterized by reduced diversity and temporal instability.^7^ During periods of disease flares, it shifts toward dysbiosis with a reduced abundance of short chain fatty acids producing obligate anaerobes such as *F. prausnitzii* and overgrowth of facultative anaerobes, particularly *E. coli*.^8,9^ However, the exact sequelae and mechanisms connecting dysbiosis with flares in IBD disease activity remain elusive. IBD patients harbor *E. coli* isolates that primarily belong to B2 and D phylogroups with virulence factors that have originally been described in extraintestinal pathogenic *E. coli*.^10–12^ Efficient horizontal gene transfer of pathogenicity islands (PAI) and high genetic plasticity in chromosomes and plasmids facilitate the rapid adaptation of *E. coli* to various ecological niches.^13,14^ *E. coli* thrives in areas of ulcerations and its DNA has been detected in 80% of CD patient’s granulomas.^15,16^ Adherent-invasive *E. coli* in CD can replicate within macrophages and induce TNF-α secretion in vitro.^17^ UC-associated *E. coli* were shown to potentiate intestinal inflammation in vivo.^18,19^ Large-scale adoption of multiplex-PCR-based GI pathogen panels identified enteric pathogens, including attaching and effacing *E. coli* (AEEC), being connected to inflammatory flares in IBD.^20,21^

AEEC are diarrheagenic *E. coli* and possess the PAI locus of enterocyte effacement (LEE) which harbors a type three secretion system (T3SS), genes coding for the intimin protein (*eae*), its translocated receptor (*tir*), together with regulators, chaperones and other effector proteins such as *espG*^22^ that are secreted into host cells. Their characteristic histopathological attaching and effacing lesions in intestinal epithelium result from cytoskeletal rearrangements due to binding of the adhesin intimin and translocated *tir*. Atypical enteropathogenic *E. coli* (aEPEC) are defined by possessing LEE, but lacking the EHEC (enterohemorrhagic *E. coli*) specific virulence factor Shiga toxin (*stx*) and the *E. coli* adherence factor bundle forming pili (*bfp*) of typical EPEC (tEPEC), which is necessary for localized adherence.^23^ aEPEC are a heterologous group of organisms that developed by repeated acquisition of LEE variants into different chromosomal backgrounds.^24^ Depending on O (somatic) and H (flagellar) antigens, EPEC can be serotyped with 12 classic O-groups originally recognized by the World Health Organization. Over 80% of aEPEC reported in the literature belong to non-classical EPEC serogroups and more than one-quarter is O non-typable.^25^ Clinically relevant EPEC serotypes have been highlighted in Supplementary Table 1. While EHEC and tEPEC are strongly associated with diarrhea, aEPEC is also prevalent in asymptomatic healthy individuals.^26,27^ Lacking Shiga toxin and the ability for localized adherence, aEPEC pathogenicity seems to be dependent on effector protein repertoire and host susceptibility. T3SS effector proteins are secreted into host cells and alter a variety of pathways. Depending on the secretome composition, the net effect can be either pro- or anti-inflammatory.^28^

The majority of aEPEC T3SS effector proteins are not located on the LEE and their biological function remains poorly understood.^29^ They cluster in PAI surrounded by transposase-like genes, suggesting horizontal acquisition.^30^ The LEE-encoded effector protein *espG* has been shown to activate P-21 activated kinases (PAK).^31^ PAKs are serine/threonine kinase effectors of small Rho GTPases Rac1/Cdc42 and orchestrate signaling cascades, involved in cytoskeletal reorganization, cell migration, wound healing, intestinal crypt homeostasis and innate immune response.^32^ PAK 1/2 kinases are involved in host–pathogen interactions and are shown to be central to microbial infections. Bacterial pathogens and their virulent effector proteins hijack host cellular signaling pathways in which PAK1 is a key player.^33,34^ Overactivation of PAK1 and PAK2 has been implicated as important drivers of colitis, providing a potential link between aEPEC infection and IBD flares.^35–37^

Here, we systematically studied the prevalence of diarrheagenic *E. coli* in IBD patients and age- and sex-matched controls without GI symptoms. We further investigated microbiota composition in aEPEC-positive UC patients and performed whole-genome sequencing of clinical aEPEC isolates, including analysis of non-LEE-effectors and strain phylogeny. Thereby, the present study enhances the understanding of this emerging opportunistic pathogen and provides potential opportunities for secondary prevention in UC.

## Material and methods

### Screening for diarrheagenic *E. coli*

A random subset of IBD patient’s stool samples collected between 2012 and 2017 for calprotectin analysis (BÜHLMANN fCAL ELISA, Switzerland) at the Department of Internal Medicine III, Medical University of Vienna, Austria (n = 630 patients) were screened for the presence of aEPEC, tEPEC, EIEC, ETEC, EAEC or EHEC using a multiplex qPCR-based approach. Twenty-eight patients had longitudinal samples. Only the earliest time point was used for analysis of prevalence and clinical parameters to prevent bias. DNA from an age- and sex-matched control cohort (n = 234 subjects) without GI symptoms was provided by the commercial gut microbiome testing company myBioma (Austria). Calprotectin comparison between groups was done with the Mann–Whitney *U* test, prevalences were compared using Fisher’s exact test. D’Agostino & Pearson omnibus normality test was applied prior to paired t test with longitudinal calprotectin values. The average time between longitudinal sample points was 9.25 months. All p-values from statistical tests in this study are two-tailed. For additional information see supplementary methods.

### Analysis of microbiota composition

16S rRNA gene amplicon sequencing of n = 25 aEPEC-pos/aEPEC-neg UC stool samples was performed as described previously. Briefly, the standard Illumina protocol and MiSeq technology were applied followed by amplicon sequence variant (ASV) analysis with DADA2^38^ and modified Rhea scripts.^39^ For taxonomic classification, SINA version 1.6.1 with the SILVA database SSU Ref NR 99 release 138 was used with default parameters. Differential abundance ASVs were analyzed using DESeq2.^40^ Raw 16S rRNA amplicon sequencing data has been submitted to ncbi under the acession number PRJNA902016.

### Isolation of AEEC from IBD stool samples

Sixty-two stool samples that showed positivity in intimin (*eae*) PCR were sent to the Austrian Agency for Health and Food Safety (AGES, Austria) for isolation of AEEC, with a success rate of 43,5%. Bacterial isolation was based on colony picking from selective agar followed by performing *eae* PCR first from pools, and if positive from single colonies. The procedere was stopped at 50 examined colonies per sample. Isolated AEEC were O- an H-serotyped by agglutination. Additionally, 10 aEPEC strains isolated from outbreaks of diarrheagenic disease were included in the analysis, and 20 strains isolated from healthy children were ordered from the Statens Serum Institut (Denmark). A list of the 57 strains used in this study can be found in Supplementary Table 2.

### In vivo AEEC pathogenicity experiments

Trans epithelial electrical resistance (TEER) experiments were performed using Caco-2 monolayers. Primary human colon epithelial cells (HCEC-1CT) were used for the assessment of pro-inflammatory signaling (IL-8 secretion and p21-kinase expression) induced by aEPEC. Pairwise comparison was performed with the Mann–Whitney *U* test and ANOVA with Dunn’s multiple comparison test for comparing multiple groups. Additional information on experimental setup and cell culture media/conditions can be found in the supplementary method section.

### In vitro biofilm formation assay

Fifty-seven *E. coli* isolates were grown on MacConkey agar for 24 hours under aerobic or anaerobic conditions, using Anaerobox and AnaeroGen sachets (Thermo Scientific, Oxoid). Single colonies were inoculated in 5 ml brain heart infusion (BHI, 37 g/L) medium with supplements (5 g/L yeast extract, 1 g/L NaHCO_3_, 1 g/L L-cysteine, 1 mg/L vitamin K1, 5 mg/L hemin) or in LB medium and grown under aerobic or anaerobic conditions for 6 hours at 37°C. Bacterial cells were diluted to an OD600 = 0.05. 100 µl of cell suspension was transferred to the U-bottom polystyrene 96-well plates (Costar) in four technical replicates. Plates were incubated at 37°C for 48 hours under aerobic or anaerobic conditions. Supernatants were removed, bacterial biofilms were fixed with 150 μL BOUIN solution (0.9% picric acid, 9% formaldehyde and 5% acetic acid) for 15 min and washed three times with 190 μL PBS. For staining, 150 μL 0.1% crystal violet solution was added for 10 min and washed three times with H_2_0. For biofilm quantification, crystal violet in dried plates was dissolved in 190 μL 30% acetic acid and the plate was placed on a shaker for 1 h. Absorbance of 1:5 dilutions was measured on an Anthos 2010 microplate reader at 595 nm and 405 nm reference wavelength. Pairwise comparison was performed with the Mann–Whitney *U* test and ANOVA with Dunn’s multiple comparison test for comparing multiple groups.

### Whole-genome sequencing and bioinformatic analysis

Bacterial DNA was extracted using a phenol chloroform-based method. Whole-genome sequencing was performed using HiSeqV4 PE125 methodology. For genome assembly, the spades pipeline was used. Assemblies were submitted to NCBI for annotation. The CFSAN SNP pipeline was used with the *E. col*i reference genome O103:H2 12009 to construct an SNP matrix with the 57 strains from this study and 348 publicly available AEEC genomes of diverse pathotypes and one *E. albertii* genome. For phylogenomic maximum likelihood inference, IQ-TREE was applied with the best-fit model automatically selected by ModelFinder.^41^ For pangenome analysis, the Roary pipeline was used with standard parameters, followed by Scoary for the identification of associations between all genes in the accessory genome and EspG2 and EspV positivity.^42^ Pangenome composition was visualized with Phandango. To investigate the presence of known virulence factors, the VFDB database was used. For the detection of novel hypothetical secreted proteins and additional secretion systems, the EffectiveDB was applied. Pairwise comparison between EspG2-pos and EspV-pos genomes was performed with the Mann–Whitney *U* test, prevalences were compared using Fisher’s exact test, with Bonferroni correction for multiple comparisons. Sequencing data and assemblies are publicly accessible at NCBI under the project number PRJNA528578. Additional information on bioinformatic analysis can be found in the supplementary method section.

### Ethics statement

The study was reviewed and approved by the ethics committee of the Medical University of Vienna (EK-Nr: 1522/2015). The study was conducted in accordance with the ethical principles expressed in the Declaration of Helsinki and the requirements of applicable federal regulations.

## Results

### Atypical EPEC correlate with disease activity in UC

**Figure 1.**
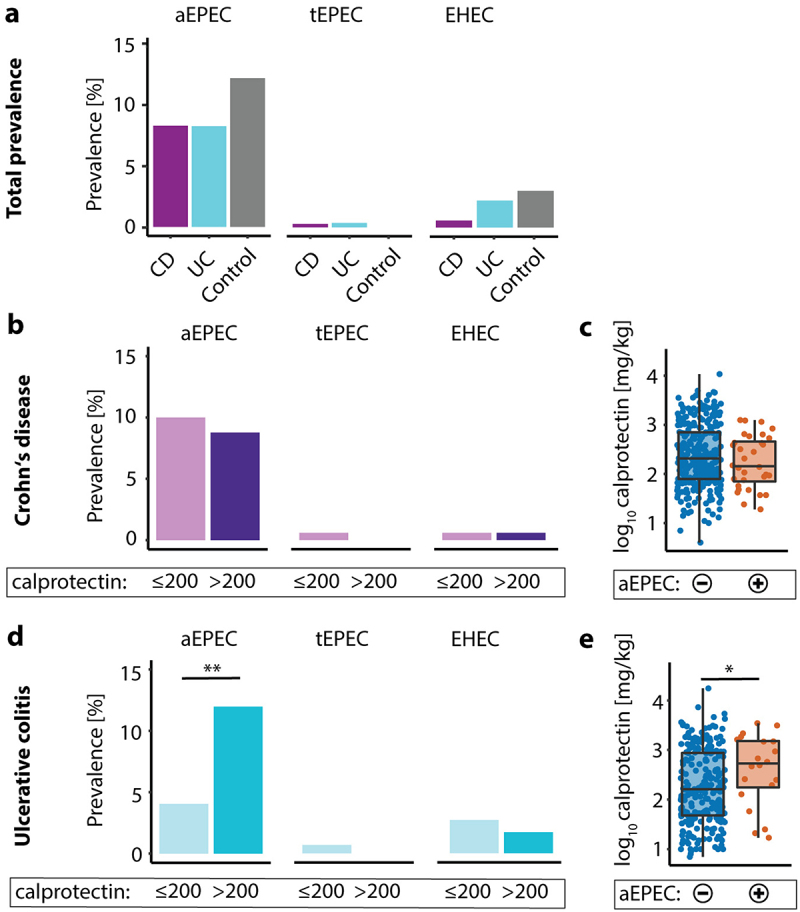
aEPEC is more prevalent in UC patients with active disease. (a) Prevalence of aEPEC, tEPEC and EHEC in CD- (purple), UC-patients (blue) and age and sex matched controls without GI-symptoms (gray). (b) Prevalence of aEPEC, tEPEC and EHEC in CD with fecal calprotectin below (light purple) and above 200 mg/kg (dark purple). (c) Fecal calprotectin in aEPEC-neg (blue) and aEPEC-pos (Orange) CD patients. (d) Prevalence of aEPEC, tEPEC and EHEC in UC with fecal calprotectin below (light blue) and above 200 mg/kg (blue). (e) Fecal calprotectin in aEPEC-neg (blue) and aEPEC-pos (Orange) UC patients. Statistical analysis: (a,b,c) Fisher’s exact text, (c,e) log_10_ transformed y-axis, Mann–Whitney *U* test, n = 356 CD, 274 UC and 234 controls; *p ≤ .05; **p ≤ .01.

We first screened fecal samples from patients with UC (n = 274), CD (n = 356) and age- and sex-matched controls without GI symptoms (n = 234) for the presence of AEEC using a multiplex qPCR-based approach. EHEC and tEPEC were rare, with less than 3% and 0.4% prevalence in all cohorts, respectively. The diarrheagenic *E. coli* subtype enteroaggregative E. coli (EAEC) had less than 4% prevalence and enterotoxigenic *E. coli* (ETEC) as well as enteroinvasive *E. coli* (EIEC) were not detectable in our cohort. aEPEC, however, could be detected in approximately 10% of samples (Figure 1a). To determine the connection between active GI inflammation and presence of aEPEC, fecal calprotectin was analyzed in the same samples.^43^ There was no association between calprotectin and aEPEC positivity in CD (Figure 1b and c). However, UC patients with GI inflammation had increased aEPEC prevalence compared to UC patients without GI inflammation (12% vs. 4%, p = .01, Figure 1d). aEPEC-positive (aEPEC-pos) UC patients exhibited median calprotectin values that were more than three times as high as aEPEC negative (aEPEC-neg) (625 vs. 162 mg/kg, p = .01, Figure 1e). Endoscopic and clinical disease activity were also higher in EPEC-pos UC patients, suggesting a link between aEPEC and flares of disease activity in UC (Table 1). In CD, there was no difference in endoscopic or clinical disease activity between aEPEC-pos and aEPEC-neg patients (Supplementary Table 2). Age, sex, disease extent, age of diagnosis, medication and smoking status were not associated with aEPEC in CD and UC (Table 1, Supplementary Table 2). Enteroaggregative *E. coli* (EAEC) did not correlate with GI inflammation (Supplementary Figure 1).

**Table 1.** Clinical parameters of aEPEC-pos UC patients.

Cohort	Variables	DEC^−^ patients (n = 550)	aEPEC^+^ patients (n = 53)	p-value^†^	
Ulcerative colitis	Total number	238	23		
	Sex (% female), n (%)	111 (47%)	8 (35%)	0.38	
	Age at sampling [years]	43 [32–56]	41 [27–57]	0.52	
	Age at diagnosis [years]	27 [23–41]	26 [19–33]	0.27	
	Proctitis (E1), n (%)	23/168 (14%)	3/16 (19%)	0.34	
	Left-sided colitis (E2), n (%)	72/168 (43%)	4/16 (25%)		
	Pancolitis (E3), n (%)	73/168 (43%)	9/16 (56%)		
	IBD related surgery, n (%)	21/238 (9%)	3/23 (13%)	0.45	
	**Endoscopically inactive disease^†^ (Mayo 0), n (%)**	**33/78 (42%)**	**2/8 (25%)**	0.04*	
	**Mild endoscopic disease^†^ (Mayo 1), n (%)**	**19/78 (24%)**	**1/8 (12%)**		
	**Moderate endoscopic disease^†^ (Mayo 2), n (%)**	**13/78 (17%)**	**0/8 (0%)**		
	**Severe endoscopic disease^†^ (Mayo 3), n (%)**	**13/78 (17%)**	**5/8 (63%)**		
	**Clinical disease activity[partial mayo score]**	**1 [0–3]**	**2 [1–5]**	0.03*	
	**Fecal calprotectin [mg/kg]**	**162 [47–864]**	**625 [178–1677]**	0.012*	
	C-reactive protein [mg/L]	0.25 [0.08–0.71]	0.32 [0.12–1.86]	0.23	
	Non-antibody-based immunotherapy, n (%)	55/207 (27%)	6/21 (29%)	0.8	
	Antibody-based immunotherapy, n (%)	51/207 (25%)	5/21 (24%)	1	
	5-ASA, n (%)	176/207 (85%)	16/21 (76%)	0.34	
	Corticosteroids, n (%)	21/207 (10%)	4/21 (19%)	0.26	
	Probiotics, n (%)	22/207 (11%)	3/21 (14%)	0.71	
	Nicotine, n (%)	18/207 (9%)	1/21 (5%)	1	

Notes: Values are presented as median with range in brackets for continuous variables or number and percentage in brackets for categorical variables. Percentages are calculated based on the actual number of patients in each group where the respective data was available. If data was not available for all subjects, the number of subjects for which the respective data was available is indicated after the backslash. The Mann–Whitney *U* test and two-sided Fisher’s exact test were used to determine p-values for continuous and categorical variables, respectively. †aEPEC positive patients vs. DEC negative patients, *p ≤ 0.05.

Abbreviations: DEC, Diarrheagenic *E. coli*; aEPEC. atypical enteropathogenic *E. coli.*

**Figure 2.**
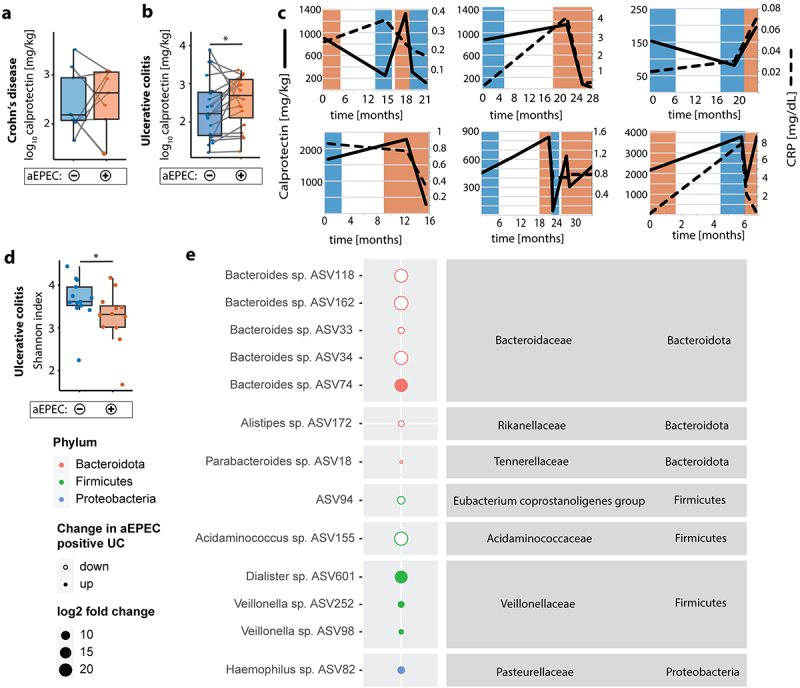
Longitudinal clinical parameters and microbiome analysis of aEPEC-pos UC patients. (a) Longitudinal fecal calprotectin in aEPEC-neg (blue) and aEPEC-pos (Orange) UC patients, samples from the same patient connected with gray lines. (b) Longitudinal fecal calprotectin in aEPEC-neg (blue) and aEPEC-pos (Orange) CD patients, samples from the same patient connected with gray lines. (c) Longitudinal trajectories of fecal calprotectin and serum c-reactive protein, with aEPEC-pos timepoints (Orange background) and aEPEC-neg timepoints (blue background). (d) UC patient’s stool bacterial diversity represented by Shannon index, aEPEC-neg (blue) and aEPEC-pos (Orange). (e) DESeq2 analysis at ASV level, aEPEC-pos vs. aEPEC-neg UC patients, Size represents fold-change, full dots represent up-regulation, empty dots down-regulation. Significant findings (p < .05 corrected for multiple comparisons) are shown. Statistical analysis: (a,b) Log_10_ transformed y-axis, ratio paired t test, n = 9 CD, 19 UC paired samples (d) Mann–Whitney *U* test, (d,e) n = 12 aEPEC-pos and 13 aEPEC-neg UC patients; *p ≤ .05.

Longitudinal analysis confirmed the link of aEPEC and GI inflammation in UC, as patients had lower calprotectin at aEPEC-neg points of time, which was not seen for CD patients (Figure 2a and b). Tracing aEPEC status over time showed transient phases of aEPEC positivity in IBD patients, suggesting re-infection or abundances below the limit of detection of a resident aEPEC (Figure 2c). 16S rRNA gene amplicon sequencing revealed reduced bacterial diversity as defined by Shannon index in aEPEC-pos UC patients compared to aEPEC-neg (Figure 2d). aEPEC-pos UC patients had increased abundances of amplicon sequencing variants (ASV) belonging to *Dialister, Haemophilus* and *Veillonella*. RDA-analysis showed a significant effect of aEPEC-positivity on microbiome composition, with fecal calprotectin following a similar gradient (Supplementary Figure 2a). ASV belonging to protein metabolizing *Acidaminococcus* were reduced in aEPEC-pos UC. Furthermore, several ASV belonging to *Bacteroides* were reduced and one was increased (Figure 2e). Adjusting the DESeq2 model for GI inflammation confirmed an increase in ASV belonging to *Haemophilus in* aEPEC-pos UC and revealed an enrichment of ASV belonging to sulfate reducing *Bilophila* and several beneficial bacteria such as Eubacterium and Subdoligranulum (Supplementary Figure 2b and c). Overall, these findings support the concept that aEPEC are correlated with flares in disease activity and microbiota dysbiosis in UC.

### *UC-associated aEPEC elicit a pro-inflammatory response* in vitro

**Figure 3.**
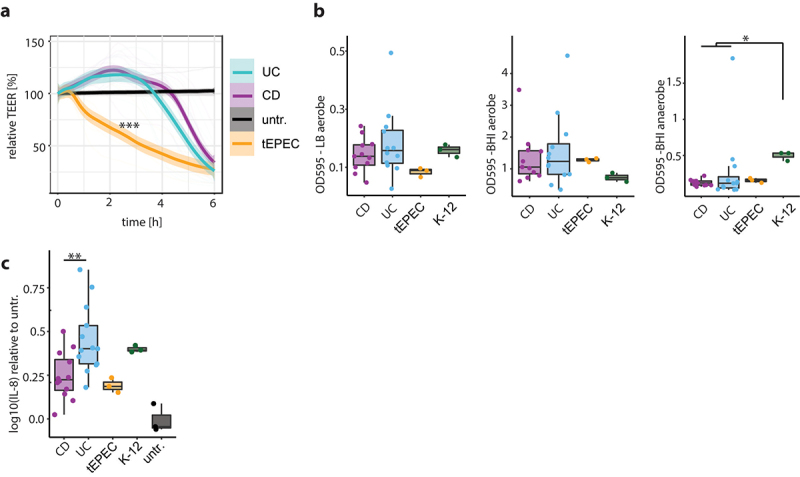
aEPEC strains from UC elicit a more pro-inflammatory in vitro response than CD. (a) Trans epithelial resistance of Caco-2 monolayers infected with aEPEC strains isolated from UC patients (blue), CD patients (purple) and reference strain tEPEC-E2348/69 (Orange), with untreated cells (black). (b) Biofilm formation assay of aEPEC strains isolated from UC patients (blue) and CD patients (purple), tEPEC-E2348/69 (Orange), *E. coli* K-12 (green) aerobic conditions with LB medium (left), aerobic conditions with BHI medium (middle) and anaerobic conditions with BHI medium (right). (c) IL-8 secretion of human primary colon epithelial cells (HCEC-1CT) infected with aEPEC strains isolated from UC patients (blue) and CD patients (purple), tEPEC-E2348/69 (Orange), *E. coli* K-12 (green) normalized to untreated cells (black). Statistical analysis: (a,b) n = 11 CD, 12 UC, (c) n = 12 CD, 13 UC, (a) two-way ANOVA with Tukey’s multiple comparisons test, (b,c) Mann–Whitney *U* test; *p ≤ .05;**p ≤ .01; ***p ≤ .001.

To investigate how aEPEC could trigger GI inflammation and if aEPEC from UC patients behave differently than aEPEC from CD patients, we performed in vitro pathogenicity experiments using strains isolated from IBD patient’s fecal samples (n = 13 UC, n = 12 CD). Disruption of epithelial tight junction (TJ) barrier is a well-defined pathomechanism of tEPEC infection, thereby inducing diarrhea. To test for TJ barrier disruption, bacteria were co-cultivated with Caco-2 monolayers, and barrier function was assessed continuously using electric cell-substrate impedance sensing (ECIS) technology. The tEPEC-E2348/69 reference strain led to a steady decrease in transepithelial electrical resistance (TEER) with a ~50% drop after 3 h. aEPEC strains from CD and UC patients showed comparable TEER responses with an initial rise of barrier function which peaked at 2 h, followed by an abrupt drop as the infection progressed (Figure 3a). Recently, bacterial biofilm formation has been implicated in IBD pathophysiology.^44^ aEPEC built strong biofilms in rich BHI media and under aerobic conditions. Biofilm formation was less pronounced with LB media without additional glucose and in anaerobic conditions. There was no difference between CD- and UC-associated aEPEC regarding biofilm formation (Figure 3b). Depending on T3SS effector proteins, EPEC can induce either a pro- or anti-inflammatory epithelial response.^24^ Thus, aEPEC strains were co-cultivated with immortalized human primary colon epithelial cells (HCEC-1CT), and IL-8 secretion was measured as a marker of pro-inflammatory signaling. In agreement with our clinical findings, UC-associated aEPEC elicited a stronger IL-8 response compared to CD-associated aEPEC (Figure 3c). tEPEC-E2348/69 attenuated IL-8 secretion, while *E. coli* K-12 induced IL-8 secretion at a comparable rate to UC-associated aEPEC (Supplementary Figure 3b). When comparing in vitro pathogenicity findings with aEPEC strains isolated from infectious diarrhea patients (n = 12) and healthy controls (n = 20), aEPEC from healthy controls led to a higher initial increase and less pronounced drop in TEER, as well as showing stronger biofilm formation under anaerobic conditions (Supplementary Figure 3a and c). *E. col*i K-12 had stronger biofilm formation than IBD-associated aEPEC under anaerobic conditions (Figure 3b). Compared to UC-associated aEPEC, aEPEC from infectious diarrhea patients had weaker biofilm formation in LB media without glucose (Supplementary Figure 3c). The majority of aEPEC strains isolated from IBD patients belonged to previously unrecognized non-classical aEPEC serotypes and there was no association between serotype and disease cohort (Supplementary Tables 1, 3). Altogether, these data indicate that UC-associated aEPEC show virulent in vitro phenotypes, resembling biofilm formation, barrier dysfunction and inflammation.

### Non-LEE effector proteins EspG2 and EspV distinguish aEPEC from CD and UC

**Figure 4.**
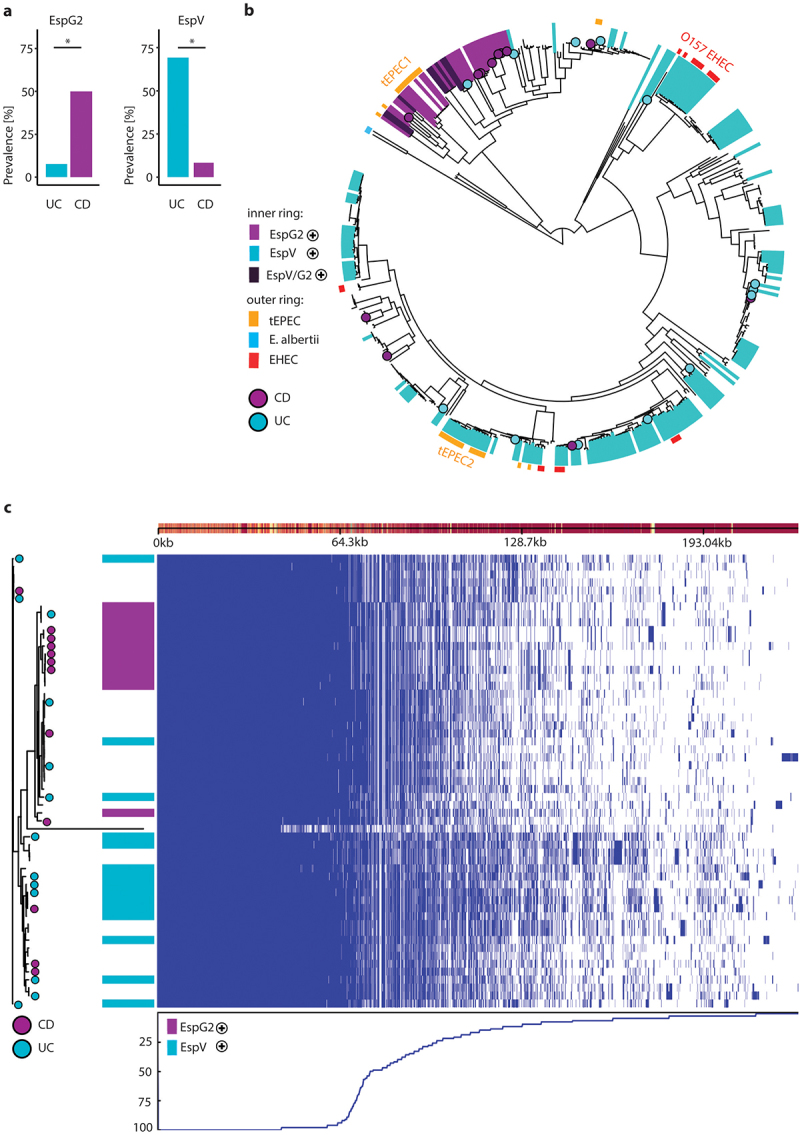
Phylogeny of AEEC and characterization of EspG2 and EspV-carrying aEPEC. (a) Prevalence of non-LEE effectors EspG2 (left) and EspV (right) in aEPEC isolates from UC- (blue) and CD-patients (purple). (b) Midpoint rooted tree constructed with 405 AEEC genomes. Strains isolated from UC- (blue circle) and CD-patients (purple circle) labeled at the tip. Inner ring depicts the presence of EspG2 (purple), EspV (blue) or both (dark purple) in the genomes. Outer ring depicts pathotype; tEPEC (Orange), E. albertii (light blue) and EHEC (red). (c) Pangenome of 57 AEEC investigated in this study, tree from alignment of 2719 orthologous proteins (left) with strains isolated from UC- (blue circle) and CD-patients (purple circle) labeled at the tip. Column depicts the presence of EspG2 (purple), EspV (blue), genome presence absence matrix (right) blue line below shows the percentage of isolates carrying a gene at each position and linearized (pan)genome, with genes displayed as rectangles above. Statistical analysis: (a) Fisher’s exact text; *p ≤ .05.

To analyze the population structure, a maximum likelihood (ML) phylogeny was constructed using a reference-based single nucleotide polymorphism matrix of aEPEC isolates from IBD patients, infectious diarrhea patients and healthy controls (total n = 57), together with 348 publicly available AEEC genomes of diverse pathotypes and one *E. albertii* genome. The isolated strains were initially identified to be aEPEC based on PCR detection of *eae* but not *bfpA* or *stx*. Analysis of sequencing data revealed one supposed aEPEC isolate from the healthy cohort to possess *stx1*. The strain was reclassified as EHEC together with another strain from the healthy cohort which was reclassified as *E. albertii* after phylogenetic analysis (Supplementary Figure 4a). The AEEC genomes clustered in 14 clonal groups (CG) containing >5 isolates which were named based on their dominant Achtman multi-locus sequence type (Supplementary Figure 4a). aEPEC evolved through multiple LEE acquisition events via horizontal gene transfer.^24^ To compare evolutionary history of LEE with the whole-genome evolution in the 57 isolated strains, ML trees were generated using aligned LEE encoded genes and a concatenated alignment of 2719 single-copy common genes (Supplementary Figure 4c). Comparison of the resulting trees points toward possible recombination events within the LEE (Extended Data Supplementary Figure 1). The LEE sequences clustered in three lineages, with the majority of strains belonging to the LEE1 and LEE3 lineages (Supplementary Figure 4d). LEE1 had more known non-LEE effector proteins than LEE3 (p < .006, two-sided Mann–Whitney *U* test with Bonferroni correction). Ninety-one percent of CD-associated aEPEC belonged to LEE3 compared to 54% of UC-associated aEPEC (Supplementary Table 4, Fisher’s exact test, p < .05). There was no association between CG and disease cohort. Intimin comprises three major (α, β and γ) and multiple minor subtypes (epsilon: ɛ, iota: ɩ and zeta: ζ) which have been linked to tissue tropism and evolutionary branches of EPEC.^45,46^ The intimin subtypes of our isolated strains were scattered across disease cohorts (Supplementary Table 3). As effector protein composition could explain the different in vitro behavior of UC- and CD-associated aEPEC, an exploratory analysis of known non-LEE effector proteins was performed using recursive partitioning. EspV was more abundant among UC-associated aEPEC strains (61% vs. 8%, Fisher’s exact: p < .05), and EspG2 was more prevalent in aEPEC strains from CD patients (50% vs. 8%, Fisher’s exact: p < .05) (Figure 4a, Supplementary Figure 5). Taken together, these findings suggest that distinct subgroups of aEPEC are detectable in UC vs. CD patients.

### EspV-positive aEPEC are more virulent than EspG2-positive aEPEC

Typical EPEC evolved multiple times within *E. coli* through independent acquisition events of LEE PAI and the EAF plasmid. Strains have traditionally been classified into two major clades EPEC1 and EPEC2, which belong to phylogroups B2 and B1, respectively.^47^ In humans, intimin α is specifically expressed by EPEC1, and intimin β has primarily been associated with EPEC2.^45^ EspG2 is a non-LEE encoded homolog of the LEE gene EspG and has been described in EPEC1.^48^ Indeed, aEPEC that harbor EspG2 clustered together in close proximity to the ‘typical’ EPEC1 clade, which also includes tEPEC-E2348/69 (bootstrap: 100%), while EspV harboring aEPEC were scattered across the tree. The only clonal group among EspG2-pos aEPEC was CG526, while EspV-pos aEPEC strains were placed in six different clonal groups (Figure 4b, Supplementary Figure 4a). All EspG2-positive (EspG2-pos) aEPEC from the 57 isolated strains had the LEE3 lineage and all intimin α possessing strains fell into this clade as well (Supplementary Figure 4d). A subgroup of EspG2-pos aEPEC were also EspV-positive (EspV-pos), however none of the 57 isolated strains possessed both genes. Eighty percent of EHEC genomes were EspV-pos compared to 40% of aEPEC strains, indicating a correlation with in vivo pathogenicity (Fisher’s exact: p < .001). To investigate further genetic differences between EspG2-pos and EspV-pos aEPEC strains, we performed pangenome analysis of the 57 isolated strains using Roary. The number of genes in the resulting pangenome continues to increase non-asymptotically with each additionally added isolate, classifying it as an open pangenome (Extended Data Supplementary Figure 2). Twenty percent (3190) of the identified genes were ‘core genes’ present in more than 95% of the isolates. ‘Accessory genes’ represented 80% of the pangenome (12895 genes). A large proportion of the accessory genomes were present in fewer than 15% of the strains (10191/12895 genes), highlighting genomic diversity and plasticity of AEEC. Visualizing the pangenome with phandango revealed fingerprint patterns of the accessory genome in EspG2-pos and EspV-pos clusters, hinting at an association of distinct genetic compositions with EspG2 and EspV positivity (Figure 4c). To identify genes associated with EspG2 and EspV we utilized the microbial pan-GWAS pipeline Scoary, which discovered 968 genes distinguishing EspG2-pos and EspV-pos strains (p < .05 after Benjamini–Hochberg correction). Gene ontology analysis linked the genes with benzene-containing compound metabolic processes and cellular response to xenobiotic stimulus (p < 7.4E-09). Among the genes co-occurring with EspV were several virulence factors, laminin-binding fimbriae (*elfD*/G), type 1 fimbriae (*fimH*) and multiple genes annotated as iron ABC transporter permeases (Extended data Table 1). The virulence factor database (VFDB) is a manually curated database for virulence factors of medically important pathogens. Using the VFDB, genomes of EspV-pos aEPEC were shown to possess more known virulence factors linked to adherence and invasion, as well as autotransporters and toxins (Table 2). Seventy-five percent of EspV-pos aEPEC had the gene encoding for hemolysin E and none for EspG2-pos. Furthermore, the EffectiveDB pipeline was applied to discover novel T3SS effector proteins based on their N-terminal signal peptide and classify if strains possess a functioning type 4 and 6 secretion system (T4SS, T6SS). EspV-pos strains had more total predicted secreted proteins, T3SS effector proteins (median T3SS: 473 vs. 383, p < .003) and T3SS chaperones. Sixty-seven percent of EspV-pos aEPEC had a functioning T6SS predicted with high confidence, compared to none of the EspG2-pos strains (Table 2). Overall, the genomic data indicate increased potential for virulence in EspV-pos aEPEC.

**Table 2.** Genomic characteristics of clinical aEPEC isolates.

Database	Variables	EspV^+^ aEPEC (n = 15)	EspG2^+^ aEPEC (n = 12)	p-value^†^
VFDB	**Adherence**	**28 (24–30)**	**21.5 (21–28)**	**0.001**
	**Autotransporter**	**4 (2–6)**	**3 (2–3)**	**0.01**
	**Invasion**	**2 (1–3)**	**3 (2–3)**	**0.034**
	Iron uptake	10 (0–18)	7 (7–21)	0.881
	**Toxin**	**1 (0–7)**	**0 (0–3)**	**0.003**
	Immune evasion	1 (0–2)	1 (0–2)	0.529
	Anti-phagocytosis	0 (0–1)	0 (0–1)	0.624
EffectiveDB	**Predicted secreted proteins**	**996 (808–1121)**	**827.5 (804–889)**	**0.0004**
	**Predicted T3SS effectors**	**473 (381–543)**	**383 (375–418)**	**0.003**
	**Conserved binding domains of T3SS chaperones**	**106 (94–117)**	**101.5 (95–106)**	**0.036**
	**Predicted T4SS effectors**	**118 (96–130)**	**102.5 (93–124)**	**0.015**
	Unique eukaryotic-like domains	28 (22–35)	29.5 (20–32)	0.435
	Nr. of proteins with eukaryotic-like domains	104 (65–131)	94.5 (59–104)	0.180
	High likelihood of functioning T3SS	15/15 (100%)	12/12 (100%)	1
	High likelihood of functioning T4SS	3/15 (20%)	2/12 (17%)	1
	**High likelihood of functioning T6SS**	**10/15 (67%)**	**0/12 (0%)**	**0.0004**

Notes: Values are presented as median with first and third quartiles in brackets for continuous variables or number and percentage in brackets for categorical variables. The Mann–Whitney *U* test and two-sided Fisher’s exact test were used to determine p-values for continuous and categorical variables, respectively. ^†^EspV positive vs. EspG2 positive strains.

### aEPEC induce PAKs and 5-ASA counteracts their pro-inflammatory stimulus

**Figure 5.**
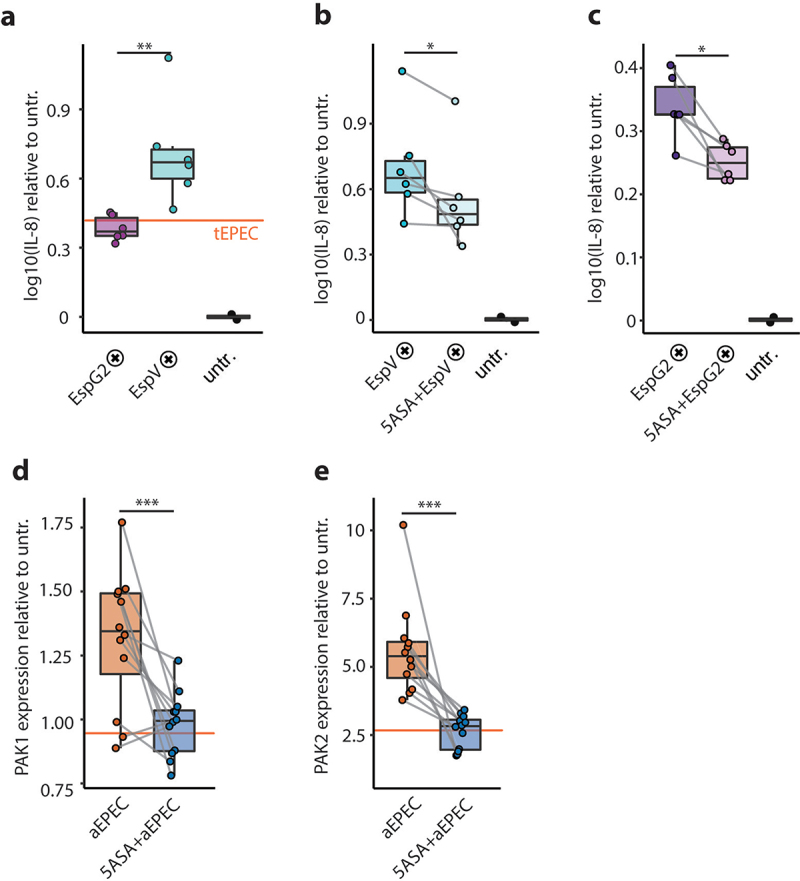
5-ASA dampens the aEPEC-induced pro-inflammatory epithelial response. (a) IL-8 secretion of human primary colon epithelial cells (HCEC-1CT) infected with EspG2- (purple) and EspV-positive (blue) aEPEC strains, normalized to untreated cells (black). (b) IL-8 secretion of primary colon epithelial cells infected with EspV-positive AEEC strains, without (blue) and with 5-ASA (light blue), normalized to untreated cells. (c) IL-8 secretion of primary colon epithelial cells infected with EspG2-positive AEEC strains, without (purple) and with 5-ASA (light purple), normalized to untreated cells. (d) PAK1 expression of primary colon epithelial cells infected with aEPEC, relative to untreated cells with (blue) and without (Orange) 5-ASA, tEPEC (Orange line). (e) PAK2 expression of primary colon epithelial cells infected with aEPEC, relative to untreated cells with (blue) and without (Orange) 5-ASA. (a-e) Values for the reference strain tEPEC-E2348/69 are visualized with an Orange line. (a-e) Data Points from the same strains are connected with a gray line. Statistical analysis: (a) unpaired t-test, (b-e) paired t-test, (a-c) Log_10_ transformed y-axis; *p ≤ .05, **p ≤ .01, ***p ≤ .001.

During infection, typical EPEC inactivates the innate immune response via various translocated effector proteins that prevent IKK-mediated phosphorylation of IκB and NF-κB, prior to TJ disruption.^49^ This results in the phenotype of watery diarrhea with a rather weak inflammatory response. In our in vitro model using co-cultivation of aEPEC with human primary colon epithelial cells, EspG2-pos aEPEC had an IL-8 response comparable to the tEPEC-E2348/69 reference strain, while the median IL-8 secretion was doubled in response to EspV-pos strains (Figure 5a). The anti-inflammatory drug mesalamine (5-ASA) is the mainstay drug for mild-to-moderate UC. 5-ASA mediated inhibition of the master regulator PAK1 contributes to attenuation of multiple signaling pathways such as Wnt/β-catenin, ERK1/2, AKT1, mTOR, NF-kB and induction of cell cycle arrest.^32,36,50^ EspG has been shown to activate PAKs, and PAK1 has recently been identified as an important driver of colitis in IBD in an integrated in vivo multiomics study.^31,35^ Therefore, we investigated the effect of 5-ASA on aEPEC induced epithelial IL-8 secretion and PAK mRNA expression. Both Il-8 production and PAK1/2 expression were increased in aEPEC infected HCEC-1CT. 5-ASA treatment reduced the IL-8 response to EspG2-pos and EspV-pos aEPEC strains, together with a reduction of PAK1 and PAK2 mRNA expression (Figure 5b-e). 5-ASA treatment reduced the IL-8 response triggered by EspV-pos aEPEC to levels of EspG2-pos strains without 5-ASA (Figure 5b). With 5-ASA treatment, PAK expression of aEPEC infected cells was comparable to tEPEC-E2348/69 infection (Figure 5d and e). Taken together, these findings support the hypothesis that 5-ASA can reduce the pro-inflammatory response elicited by EspV-pos aEPEC in UC patients.

## Discussion

The prevalence of aEPEC has surpassed tEPEC, with aEPEC being detected in 95–99% of The EPEC-positive samples in industrialized countries. In our study cohort, we detected aEPEC in ~10% and tEPEC in 0.4% of stool samples, which is in the range of other European studies.^26,51^ The presence of aEPEC correlated with flares in UC disease activity as determined by fecal calprotectin, endoscopic- and clinical-Mayo scores. Prevalence of aEPEC was similar in UC patients with active disease and health controls but halved in UC patients in remission. UC patients might represent a vulnerable population due to mutations in pathways involved in immune–microbiota interactions and intestinal barriers.^1^ Comparing the microbiome composition of aEPEC-pos and aEPEC-neg UC samples, we detected reduced microbial diversity and an enrichment of ASVs belonging to taxa that also includes opportunistic pathogens *Dialister, Haemophilus* and *Veillonella*. Correcting for calprotectin in differential abundance analysis pointed toward an independent influence of aEPEC on microbiome composition. *Haemophilus* is typical for the oral flora, and *Veillonella* is an important biofilm initiator.^52^ Mucosal biofilms have recently been shown to be correlated with dysbiosis and inflammation in IBD.^44^ We found that aEPEC isolates from both UC and CD formed biofilms, especially under aerobic conditions. However, aEPEC isolated from healthy controls formed biofilms under anaerobic conditions. This suggests that aEPEC isolated from IBD patients and healthy subjects differ in their adaptation to environmental parameters such as oxygen tension, which is likely a consequence of dysbiosis.

Compared to tEPEC, aEPEC persists longer in the intestine, which could be due to inhibition of epithelial apoptosis and its lack of localized adhesion.^53^ We detected aEPEC positivity over several months in IBD patients together with cycles of reoccurrence. In our in vitro model, aEPEC were able to induce PAKs which are implicated in IBD pathogenesis and treatment with 5-ASA dampened PAK expression as well as IL-8 secretion. We have previously shown that PAK1 is overexpressed in IBD and is associated with increased cell survival.^37^ Thus, activation of PAK signaling could contribute to intestinal persistence of aEPEC.

Isolated aEPEC strains from IBD patients showed in vitro phenotypes resembling diarrhea with increased paracellular flux after 4–6 hours of infection. Decreased TEER by aEPEC indicates compromised epithelial barrier which can be restored by 5-ASA.^54^ Whether aEPEC infections precede and trigger IBD flares or aEPEC just thrives in an inflamed environment still remains to be determined. These results suggest that prolonged aEPEC infection could tip microbiota homeostasis and contribute to diarrhea and inflammation in UC patients. It is likely that aEPEC strains promote inflammation as a favorable ecological niche where they can outcompete commensals due to an abundance of virulence factors.

Human volunteer studies found that, contrary to tEPEC harboring *bfp*, the potential to cause diarrhea varies between different aEPEC strains and subjects.^55–57^ tEPEC is known to compromise epithelial barrier and attenuate IL-8 secretion *in vitro*.^49^ In contrast, aEPEC induced a stronger pro-inflammatory stimulus and less pronounced barrier defect which could be explained by the secretion of different effector proteins. aEPEC have a highly diverse virulence factor and effector protein repertoire due to evolution via repeated acquisition of LEE PAI variants and overall genetic plasticity.^24^ It has been previously suggested that differences in the effector protein arsenal could explain the heterogeneous clinical phenotypes of aEPEC infection.^25,26,51^ We showed that aEPEC from UC patients elicited a stronger epithelial IL-8 response than aEPEC from CD, which behaved more like tEPEC. Virulence mechanisms and human target proteins of EspV are still elusive; however, their expression in yeast results in a dramatic increase in cell size and irreversible growth arrest.^58^ Our analysis revealed that EspV-pos aEPEC were associated with UC and compared to EspG2-pos, had more virulence factors, including hemolysin E, adhesins, iron transporters and a T6SS combined with an elevated IL-8 response in vitro. Supporting the hypothesis of increased virulence potential, protein homology predicted an abundance of non-LEE T3SS effector proteins with unknown function that were enriched in EspV-pos aEPEC. The non-LEE effector protein EspG2 was associated with aEPEC from CD and could distinguish a phylogenetic aEPEC clade related to EPEC1. None of the aEPEC strains isolated from infectious diarrhea patients and just one of the strains from UC patients possessed EspG2. The preference of more virulent EspV-positive aEPEC for UC patients warrants further investigation. It might be explained by depleted mucin production, preexisting low-grade colonic inflammation or altered microbiome diversity.^8,59^

The modest sample size of our isolated strains is a limitation of this study. Possible sources of bias in the analysis are potential differences in sample storage between IBD and control cohort and age difference of healthy children vs. adult IBD patients for isolated strains. *E. coli* K-12 is known to disrupt the epithelial barrier and induces IL-8 secretion via TLR signaling in vitro.^60–62^ Additional in vivo experiments, including mutant and non-aEPEC commensal strains isolated from controls, should be performed to establish EspG2 and EspV as bona fide genes for aEPEC virulence. Furthermore, detailed longitudinal studies could uncover the exact sequelae of aEPEC infection and the onset of inflammation in UC. aEPEC isolated in this study primarily belong to nonclassical EPEC serotypes that have not been recognized clinically. EspG2, EspV and the identified serotypes could serve as targets to distinguish aEPEC strains with different pro-inflammatory potentials in intestinal inflammation. *EspG2, espG* and *virA* from *Shigella flexneri* are structural homologies.^63^ Future studies are vital to establish if EspG2 is just a marker of less co-occurring virulence factors in aEPEC genomes or alters activation of pro-inflammatory signaling pathways such as PAK.

These results imply that EspV-pos aEPEC not only thrive in the niche of an inflamed GI environment but can also induce epithelial inflammation via induction of IL-8 secretion and PAK expression. The ability of aEPEC to generate disease depends on the susceptibility of the infected person, thus making aEPEC an opportunistic pathogen. Our findings suggest that UC patients with their disturbed immune–microbiota axis and less resilient microbiota might represent such a vulnerable population. Limiting the contact with aEPEC might thus contribute to secondary prevention in UC. In any case, stool samples should be screened for aEPEC in patients with flares of UC.

## Supplementary Material

Supplemental Material
