# Supplementary material for: Atypical enteropathogenic E. coli are associated with disease activity in ulcerative colitis
Source: Gut Microbes. 2022 Nov 22;14(1):2143218. doi: 10.1080/19490976.2022.2143218 (PMC9704410; doi:10.1080/19490976.2022.2143218)
Supplement: Supplemental Material [file KGMI_A_2143218_SM2463.zip › Supl_Material_Methods_revision2_clean.docx]

# Supplementary Material and Methods

## Screening for diarrheagenic *E. coli*

DNA from IBD patients was extracted using the QIAmp DNA stool Mini Kit (Qiagen, Netherlands), concentration of dsDNA was measured using the Quant-iT PicoGreen dsDNA Kit (Invitrogen, USA). DNA for an age and sex-matched control cohort was provided by the commercial gut microbiome testing company myBioma (Austria). Control subjects provided a questionnaire and reported no GI disease (including e.g. IBD, IBS, small intestinal overgrowth, celiac disease, gastritis, stomach ulcers, reflux, diverticula disease, cancer hemorrhoids or liver pathosis). Multiplex qPCR was performed using well established target genes, primers, probes, positive controls for the different types of diarrheagenic *E. coli* (supplementary table 4), the TaqPath ProAmp Multiplex Master Mix (Qiagen, Netherlands), 5 ng of input DNA, and a 7500 Fast dx Real-Time PCR instrument (Thermo Fisher Scientific, USA). aEPEC positive samples were defined as showing a amplification for eae but not stx or bfp. Dilutions of DNA extracted from the aEPEC isolate 70-09 provided by the Austrian Agency for Health and Food Safety (AGES, Austria) has been spiked in DNA extracted from human stool to determine the limit of detection. Limit of detection was approx. ~1500 genomes / mL with a linear dynamic range of over at least 6 Log_10_. qPCR efficiency for the detection of different AEEC was in the range of 1+/-0.1. Variables as disease extent, location, clinical and laboratory disease activity, as well as medication were collected from the in-house patient database of the general hospital of Vienna. A cutoff of 200 mg/kg fecal calprotectin was used to determine active and inactive colonic disease.

## Isolation of AEEC from IBD stool samples

62 stool samples which showed positivity in intimin (*eae*) PCR were sent to the Austrian Agency for Health and Food Safety (AGES, Austria) for isolation of AEEC, with a success rate of 43,5 percent. Bacterial isolation was based on colony picking from selective agar followed by performing *eae* PCR first from pools, and if positive from single colonies. The procedere was stopped at 50 examined colonies per sample. Isolated AEEC were O- an H-serotyped by agglutination. Additionally, ten aEPEC strains isolated from outbreaks of diarrheagenic disease were included in the analysis and twenty strains isolated from healthy children were ordered from the Statens Serum Institut (Denmark). A list of the 57 strains used in this study can be found in supplementary table 2.

## *In vivo* AEEC pathogenicity experiments

Trans epithelial electrical resistance (TEER) experiments were performed using Caco-2 monolayers grown on 8W10E chamber plates for real time Electric Cell-substrate Impedance Sensing (ECIS) analysis with a ZTheta machine (Applied BioPhysics, USA). The plates were incubated with 200 µL freshly prepared 10 mM cysteine, followed by a washing step and incubation with 300 μl, 35 μg/ml collagen solution at room temperature. Ten thousand cells in DMEM with 20% FBS and 1% antibiotics (penicillin/streptomycin, P/S) were seeded per well and grown for five days, where they reached a stable plateau in TEER, measured at 500 Hz. To activate effector protein transcription, 50 µL of overnight lysogeny broth cultures were added to 5 mL DMEM without supplements followed by incubation for 4 hours in a cell culture incubator (5% CO_2_, 95% relative humidity, and 37°C). Two hours before the experiment, monolayers were washed and media replaced with pre-warmed DMEM without supplements or P/S. 100 activated bacteria per host cell were added to the wells and TEER was measured continuously every 5 minutes for six hours. Samples were prepared in duplicates and two-way ANOVA with Tukey’s multiple comparisons test was used to assess significance. Graphs were generated in R with the ggplot package and loess smoothing (span=0.1).

Primary human colon epithelial cells, HCEC-1CT (obtained from Jerry W. Shay and Andres I. Roig, University of Texas, Dallas), were cultured in basal X media (DMEM: M199, 4:1; GIBCO, Germany), supplemented with epidermal growth factor (20 ng/mL; BD Biosciences, Germany), hydrocortisone (1 mg/mL; Sigma, Germany), insulin (10 mg/mL), transferrin (2 mg/mL), sodium selenite (5 nM; all from Gibco, Life Technologies GmbH, Germany), 2% cosmic calf serum (HyClone, Germany), and gentamicin sulfate (50 mg/mL; Sigma). Bacteria were activated as described above and two hours before the experiment, cells were washed and media replaced with basal X media without gentamicin, cosmic calf serum or hydrocortisone with or without the addition of 5 mM 5-ASA (99.9% pure; a generous gift from Shire Inc., Eysins, Switzerland). 50 activated bacteria per host cell were added to the supernatant and plates were spinned at 500x g to start the infection simultaneously. After 4 hours of infection the supernatant was frozen immediately at -80°C and cells were incubated with TRIzol (Thermo Fisher scientific, USA). RNA was extracted using TRIzol, complementary DNA was synthesized using the High-Capacity Complementary DNA Reverse Transcription Kit (Applied Biosystems, USA) and RT-PCR was performed on a 7500 Fast Real-Time PCR instrument (Thermo Fisher scientific, USA) with beta actin as reference gene. Primers for PAK1 and PAK2 were ordered from Qiagen (PAK1 Qiagen (Cat# QT00068306 and QT00093695 respectively). Secreted IL-8 was measured in the supernatant using the Human IL-8/CXCL8 Quantikine ELISA Kit (R&D systems, USA). 96-well plates and triplicates were used for the first set of experiments with IL-8 analysis and 24-well plates with duplicates were used for the second set of experiments with IL-8 analysis, 5-ASA treatment and RNA extraction. Pairwise comparison was performed with Mann-Whitney U test and ANOVA with Dunn’s multiple comparisons test for comparing multiple groups.

## Whole genome sequencing and bioinformatic analysis

Bacterial DNA was extracted using a phenol chloroform based method. DNA was sheared with a S220 Focused-ultrasonicator (Covaris, UK). Shearing intensity and time was adjusted for each type of sample using a time series and capillary electrophoresis to achieve a fragment size of 200-800 bp. Library preparation was done with a NEBNext kit with dual indexing (NEB, Germany) and a size selection of 300-800 bp. Sequencing was performed on an Illumina HiSeqV4 PE125. For genome assembly the spades pipeline was used. Assemblies were submitted to ncbi for annotation. The CFSAN SNP pipeline was used with the *E. col*i reference genome O103:H2 12009 to construct a SNP matrix with the 57 strains from this study and 348 publicly available AEEC genomes of diverse pathotypes and one *E. albertii* genome. For phylogenomic maximum likelihood inference IQ-TREE was applied with the best-fit model automatically selected by ModelFinder. For CG analysis the RAMI cutoff was optimized to differentiate between the O157 EHEC (CG11) lineage and the aEPEC CG335 lineage. The phylogenetic trees for concatenated alignments of LEE encoded genes and concatenated alignments of all single-copied common genes were constructed using RaxML with standard parameters. The genes were assigned to orthologous groups using the ProteinOrtho tool v.6 with parameters cov = 75 and identity = 85; The orthogroups were aligned using Muscle v. 3.8.1551. The comparison of phylogenetic trees was performed using Python package ete3. Presence of *bfpA*, *stx* and known non-LEE effector proteins was assessed using ARIBA for assembly with reference sequences (Extended data table 2). Exploratory analysis of the association of non-LEE effector proteins with disease cohort was performed with R and the package rpart. Known non-LEE effector composition of 80% of randomly selected strains was used to build the model and the remaining 20% to estimate accuracy. For pangenome analysis the Roary pipeline was used with standard parameters, followed by Scoary for the identification of associations between all genes in the accessory genome and EspG2 and EspV positivity. Pangenome composition was visualized with Phandango. To investigate the presence of known virulence factors the VFDB database was used. For detection of novel hypothetical secreted proteins and additional secretion systems the EffectiveDB was applied. Pairwise comparison between EspG2-pos and EspV-pos genomes was performed with Mann-Whitney U test, prevalences were compared using Fisher's exact test, with Bonferroni correction for multiple comparisons. Calprotectin groups (<100, 100-300 and >300 mg/kg) have been added in the adjusted DSeq2 model to account for inflammation. Sequencing data and assemblies are publicly accessible at ncbi under the project number PRJNA528578.
