# Supplementary material for: Atypical enteropathogenic E. coli are associated with disease activity in ulcerative colitis
Source: Gut Microbes. 2022 Nov 22;14(1):2143218. doi: 10.1080/19490976.2022.2143218 (PMC9704410; doi:10.1080/19490976.2022.2143218)
Supplement: Supplemental Material [file KGMI_A_2143218_SM2463.zip › Suppl_Figures_Tables_revision2_clean.docx]

**Atypical Enteropathogenic *E. coli* are Associated with disease activity in Ulcerative Colitis**

Maximilian Baumgartner, Rebecca Zirnbauer, Sabine Schlager, Daniel Mertens, Nikolaus Gasche, Barbara Sladek, Craig Herbold, Olga Bochkareva, Vera Emelianenko, Harald Vogelsang, Michaela Lang, Anton Klotz, Birgit Moik, Athanasios Makristathis, David Berry, Stefanie Dabsch, Vineeta Khare, Christoph Gasche

**Supplementary figures**

**Figure S1: Prevalence of EAEC, EIEC and ETEC in IBD.**

**Figure S2: Effect of calprotectin in microbiome analysis of aEPEC-pos UC patients.**

**Figure S3: aEPEC *in vitro* pathogenicity experiments using human cell lines.**

**Figure S4: Phylogeny of AEEC, non-LEE effector protein composition and LEE subtypes.**

**Figure S5: Known non-LEE effector protein recursive partitioning.**

**Supplementary tables**

**Table S1: Clinically relevant EPEC serotypes.**

**Table S2: Clinical characteristics of aEPEC*^+^* CD patients**.

**Table S3: Serotype, Intimin-, and LEE-subtype, clonal group and hemolysin positivity of strains used in this study.**

**Table S4: LEE-subtypes and clonal groups of AEEC from different disease cohorts.**

**Table S5: Primer and probes used for qPCR.**

**Figure legends**

**Suppl. Figure 1.: Prevalence of EAEC, EIEC and ETEC in IBD. (a)** Prevalence of diarrheagenic *E. coli* strains: Enteroaggregative *E. coli* (EAEC), Enteroinvasive *E. coli* EIEC and Enterotoxigenic *E. coli* (ETEC) in CD with fecal calprotectin below (light purple) and above 200 mg/kg (dark purple). **(b)** Prevalence of AEEC, EIEC and ETEC in CD with fecal calprotectin below (light purple) and above 200 mg/kg (dark purple). Statistical analysis: **(a,b)** Fisher’s exact text, n=356 CD and 274 UC.

**Suppl. Figure 2: Effect of inflammation on microbiome composition. (a)** RDA-analysis of EPEC-pos vs. EPEC-neg UC patients with overlay of fecal calprotectin [mg/kg] as gradient. **(b)** UC patient’s stool bacterial diversity represented by Shannon index, <200 mg/kg (blue) and >200 mg/kg calprotectin (orange). **(c)** DESeq2 analysis at ASV level incorporating calprotectin data, aEPEC-pos vs. aEPEC-neg UC patients, Size represents fold-change, full dots represent up-regulation, empty dots down-regulation. Significant findings (p<0.05 corrected for multiple comparisons) are shown. **Statistical analysis:** (a) ANOVA, (b) Mann Whitney U test, (a) n=12 aEPEC-pos and 13 aEPEC-neg UC patients, (b,c) n=13 <200 mg/kg and n=13 >200 mg/kg calprotectin

**Suppl. Figure 3.: aEPEC *in vitro* pathogenicity experiments using human cell lines. (a)** Trans epithelial resistance of Caco-2 monolayers infected with aEPEC strains isolated from UC patients (blue), CD patients (purple), diarrhea outbreaks (diar. yellow), healthy children (healthy, gray), *E. coli* K-12 (green) and reference strain tEPEC-E2348/69 (orange), with untreated cells (black). **(b)** IL-8 secretion of human primary colon epithelial cells (HCEC-1CT) infected with aEPEC strains isolated from UC patients (blue), CD patients (purple), diarrhea outbreaks (diar. yellow), healthy children (healthy, gray), tEPEC-E2348/69 (orange), normalized to untreated cells (black). **(c)** Biofilm formation assay of aEPEC strains isolated from UC patients (blue), CD patients (purple), diarrhea outbreaks (diar. yellow), healthy children (healthy, gray), tEPEC-E2348/69 (orange), *E. coli* K-12 (green), aerobic conditions with LB medium (left), aerobic conditions with BHI medium (middle) and anaerobic conditions with BHI medium (right). Statistical analysis: **(a)** two-way ANOVA with Tukey’s multiple comparisons test, n= 11 CD, 12 UC, 12 diar., 10 healthy, **(b,c)** ANOVA with Dunn's multiple comparisons test, **(b)** n= 12 CD, 13 UC, 12 diar., 20 healthy, **(c)** n= 11 CD, 12 UC, 12 diar., 20 healthy; *p≤0.05, **p≤0.01, ***p≤0.001

**Suppl. Figure 4.: Phylogeny of AEEC, non-LEE effector protein composition and LEE subtypes. (a)** Midpoint rooted tree constructed with 405 AEEC genomes. Inner ring depicts clonal group named after the dominant Achtman multi-locus sequence type. Outer ring depicts pathotype; tEPEC (orange), E. albertii (light blue) and EHEC (red). Clades with bootstrap values above 90 % have been colored red. **(a,c,d)** Strains isolated from UC- (blue circle) and CD-patients (purple circle), diarrhea outbreaks (diar., yellow), healthy children (healthy, gray) labeled at the tip. **(b)** Prevalence of non-LEE effectors EspV (left) and EspG2 (right) in AEEC isolates from UC- (blue), CD-patients (purple), diarrhea outbreaks (diar., yellow) and healthy children (healthy, gray). **(c)** Phylogenetic tree from alignment of 2719 orthologous proteins and presence of known LEE-effector proteins (blue squares). **(d)** Phylogeny based on aligned LEE encoded genes distinguishes three major LEE subtypes, presence of known LEE-effector proteins (blue squares).

**Suppl. Figure 5.: Non-LEE effector protein recursive partitioning.** Known non-LEE effector protein based decision tree created with the R package ‘rpart’, to distinguish AEEC strains isolated from Crohn’s disease (CD, purple), Ulcerative colitis (UC, blue), healthy children (H, gray) and diarrhea outbreaks (D, orange),, relative abundance of each disease cohort is depicted in the boxes at each step of the decision tree, 80 % of randomly selected data was used to build the tree and the remaining 20 % to estimate accuracy.

**Suppl. Table 1.: Clinically relevant EPEC serotypes.**

| **Classical EPEC serotypes** | | **Newly recognized EPEC serotypes** | |
| --- | --- | --- | --- |
| O group | H antigen | O group | H antigen |
| **O26** | **H-; H11** | O39 | H- |
| **O55** | **H**-; H6; **H7**; H34 | **O51** | **H-; H40; H49** |
| O86 | H-; H8; H34 | O88 | H-; H25 |
| **O111** | **H-**; H2; H7; H8; **H9; H25** | O103 | H2 |
| O114 | H-; H2 | O127 | H-; H6 |
| **O119** | **H-; H2**; H6 | **O145** | **H-; H28; H34**; H45 |
| O125ac | H-; H6; H21 | O157 | H-; H8; H16; H45 |
| O126 | H-; H2; H12; H21; H27 |  |  |
| O127 | H-; H4; H6; H9; H21; H40 |  |  |
| O128ab | H-; H2; H7; H12 |  |  |
| O142 | H-; H6; H34 |  |  |
| O158 | H-; H23 |  |  |

Adapted from Pathogenic Escherichia coli: molecular and cellular microbiology (Motabito – Caister Academic Press – 2014, Table 1.1). Common atypical EPEC serotypes have been highlighted in bold.**Suppl. Table 2.: Clinical characteristics of aEPEC*^+^* CD patients**.

| **Cohort** | **Variables** | **DEC^-^ patients** | | | **aEPEC^+^ patients** | | **p-value^†^** | |
| --- | --- | --- | --- | --- | --- | --- | --- | --- |
| **Crohn’s disease** | Total number, n | 312 | |  | 30 | |  |  |
|  | Sex (% female), n (%) | 154 | (51%) |  | 21 | (70%) | 0.06 |  |
|  | Age at sampling [years] | 41 | [31-52] |  | 39 | [28-48] | 0.41 |  |
|  | Age at diagnosis [years] | 26 | [21-33] |  | 27 | [22-35] | 0.69 |  |
|  | Ileal Crohn’s (L1), n (%) | 103/249 | (41%) |  | 11/23 | (48%) | 0.76 |  |
|  | Colonic Crohn’s (L2), n (%) | 62/249 | (25%) |  | 7/23 | (30%) |  |  |
|  | Ileocolonic Crohn’s (L3), n (%) | 76/249 | (31%) |  | 5/23 | (22%) |  |  |
|  | Upper GI involvement (L4), n (%) | 8/249 | (3%) |  | 0/23 | (0%) |  |  |
|  | Inflammatory disease (B1), n (%) | 10/147 | (7%) |  | 1/10 | (10%) | 0.76 |  |
|  | Stricturing disease (B2), n (%) | 47/147 | (32%) |  | 3/10 | (30%) |  |  |
|  | Penetrating disease (B3), n (%) | 90/147 | (61%) |  | 6/10 | (60%) |  |  |
|  | IBD related surgery, n (%) | 172/312 | (55%) |  | 15/30 | (50%) | 0.7 |  |
|  | Endoscopically inactive disease^†^ (SES 0), n (%) | 45/125 | (36%) |  | 2/7 | (29%) | 0.9 |  |
|  | Mild endoscopic disease^†^ (SES 1), n (%) | 33/125 | (27%) |  | 3/7 | (43%) |  |  |
|  | Moderate endoscopic disease^†^ (SES 2), n (%) | 25/125 | (20%) |  | 1/7 | (14%) |  |  |
|  | Severe endoscopic disease^†^ (SES 3), n (%) | 22/125 | (18%) |  | 1/7 | (14%) |  |  |
|  | Clinical disease activity [Harvey-Bredshaw Index] | 3 | [1-6] |  | 3 | [1-5] | 0.82 |  |
|  | Fecal calprotectin [mg/kg] | 209 | [80-730] |  | 144 | [55-458] | 0.22 |  |
|  | C-reactive protein [mg/L] | 0.28 | [0.11-0.83] |  | 0.31 | [0.1-0.65] | 0.74 |  |
|  | non-antibody-based immunotherapy, n (%) | 94/263 | (36%) |  | 8/28 | (29 %) | 0.54 |  |
|  | antibody-based immunotherapy, n (%) | 102/263 | (39%) |  | 15/28 | (54%) | 0.16 |  |
|  | 5-ASA, n=263/28, n (%) | 147/263 | (56%) |  | 14/28 | (50%) | 0.56 |  |
|  | Corticosteroides, n (%) | 32/263 | (12%) |  | 3/28 | (11%) | 1 |  |
|  | Probiotics, n (%) | 24/263 | (9%) |  | 3/28 | (11%) | 0.73 |  |
|  | Nicotine, n (%) | 64/263 | (24%) |  | 6/28 | (21%) | 0.82 |  |

DEC, Diarrheagenic *E. coli*; aEPEC. atypical enteropathogenic *E. coli*

Values are presented as median with range in brackets for continuous variables or number and percentage in brackets for categorical variables. Percentages are calculated based on the actual number of patients in each group where the respective data was available. If data was not available for all subjects, the number of subjects for which the respective data was available is indicated after the backslash. Mann-Whitney U test and two-sided Fisher’s exact test were used to determine p-values for continuous and categorical variables, respectively.

† aEPEC positive patients vs. DEC negative patients

**Suppl. Table 3.: Serotype, Intimin-, and LEE-subtype, clonal group and hemolysin positivity of strains used in this study**

| **EPEC#** | **Cohort** | **EspG2** | **EspV** | **LEE** | **CG** | **Intimin** | **hlyE/clyA** | **Serotype** |
| --- | --- | --- | --- | --- | --- | --- | --- | --- |
| EPEC7 | CD | 0 | 0 | 3 |  | gamma | 0 | O84:H14 |
| EPEC10 | CD | 1 | 0 | 3 |  | alpha | 0 | Orough:Hrough |
| EPEC11 | CD | 0 | 0 | 3 | 28 | beta | 0 | O96:H7 |
| EPEC12 | CD | 0 | 0 | 3 |  | gamma | 0 | O146:HNM |
| EPEC13 | CD | 0 | 1 | 1 | 21/29 | beta | 1 | O26:HNM |
| EPEC16 | CD | 1 | 0 | 3 | 526 | iota | 0 | O145:Hrough |
| EPEC17 | CD | 1 | 0 | 3 | 526 | iota | 0 | O145:Hrough |
| EPEC19 | CD | 0 | 0 | 3 |  | epsilon | 0 | O121:H45 |
| EPEC21 | CD | 0 | 0 | 3 | 10 | gamma | 0 | Orough:H40 |
| EPEC23 | CD | 1 | 0 | 3 |  | alpha | 0 | O132:H34 |
| EPEC24 | CD | 1 | 0 | 3 |  | alpha | 0 | Orough:Hrough |
| EPEC59 | CD | 1 | 0 | 3 | 526 | iota | 0 | O145:H34 |
| EPEC1 | UC | 0 | 1 | 1 | 10 | epsilon | 0 | Orough:H40 |
| EPEC2 | UC | 0 | 0 | 1 | 3 | beta | 0 | Orough:H2 |
| EPEC3 | UC | 0 | 1 | 1 |  | epsilon | 0 | O152:H38 |
| EPEC4 | UC | 0 | 1 | 3 |  | zeta | 0 | Orough:H45 |
| EPEC5 | UC | 0 | 0 | 3 |  | beta | 0 | O33:H6 |
| EPEC6 | UC | 0 | 0 | 3 |  | beta | 1 | Orough:H6 |
| EPEC8 | UC | 0 | 1 | 2 |  | gamma | 1 | O145:H28 |
| EPEC9 | UC | 0 | 1 | 1 | 21/29 | beta | 0 | O26:H11 |
| EPEC14 | UC | 0 | 1 | 1 |  | epsilon | 0 | O157:H16 |
| EPEC15 | UC | 0 | 1 | 3 |  | gamma | 1 | O103:H25 |
| EPEC18 | UC | 1 | 0 | 3 |  | alpha | 0 | O114:Hrough |
| EPEC20 | UC | 0 | 1 | 3 | 29 | gamma | 0 | O118:HNM |
| EPEC58 | UC | 0 | 0 | 3 | 10 | gamma | 0 | O71:H40 |
| EPEC22 | diarrhea | 0 | 1 | 3 | 517 | epsilon | 0 | O88:H25 |
| EPEC25 | diarrhea | 0 | 1 | 3 | 28 | beta | 0 | O33:H6 |
| EPEC26 | diarrhea | 0 | 0 | 2 | 335 | gamma | 0 | O55:H7 |
| EPEC27 | diarrhea | 0 | 0 | 3 |  | beta | 0 | O8:H14 |
| EPEC28 | diarrhea | 0 | 0 | 3 | 28 | beta | 0 | O113:H6 |
| EPEC29 | diarrhea | 0 | 0 | 1 | 3 | beta | 0 | O128abc:H2 |
| EPEC30 | diarrhea | 0 | 0 | 3 |  | gamma | 1 | O76:H7 |
| EPEC31 | diarrhea | 0 | 0 | 3 |  | gamma | 1 | O76:H7 |
| EPEC32 | diarrhea | 0 | 0 | 3 | 10 | gamma | 0 | Orough:H40 |
| EPEC33 | diarrhea | 0 | 1 | 2 | 11 | gamma | 1 | O157:HNM |
| EPEC34 | diarrhea | 0 | 1 | 1 |  | beta | 1 | O177:HNM |
| EPEC35 | diarrhea | 0 | 0 | 2 | 335 | gamma | 0 | O55:Hrough |
| EPEC36 | healthy | 0 | 0 | 3 | 28 | beta | 1 | O168:H6 |
| EPEC37 | healthy | 0 | 0 | 1 | 40 | beta | 0 | O109,OX182:H21 |
| EPEC38 | healthy | 0 | 0 | 3 |  | beta | 0 | O33:H12 |
| EPEC39 | healthy | 0 | 0 | 3 |  | beta | 0 | O40:H6 |
| EPEC40 | healthy | 0 | 1 | 3 | 29 | gamma | 0 | O39,70:H11 |
| EPEC41 | healthy | 1 | 0 | 3 |  | alpha | 0 | O51:H49 |
| EPEC42 | healthy | 0 | 0 | 3 |  | alpha | 0 | O179:H31 |
| EPEC43 | healthy | 0 | 0 | 3 | 28 | beta | 0 | O+:H6 |
| EPEC44 | healthy | 0 | 0 | 3 | 10 | gamma | 0 | O2:H40 |
| EPEC45 | healthy | 1 | 0 | 3 | 526 | iota | 0 | O145:Hru |
| EPEC46 | healthy | 0 | 0 | 1 |  | beta | 0 | O168:H- |
| EPEC47 | healthy | 1 | 0 | 3 | 526 | iota | 0 | O145:H34 |
| EPEC48 | healthy | 0 | 0 | 3 | 40 | gamma | 0 | O153:H21 |
| EPEC49 | healthy | 0 | 0 | 3 |  | beta | 0 | O33:H6,12 |
| EPEC50 | healthy | 0 | 0 | 3 | 28 | beta | 0 | O113:H6 |
| EPEC51 | healthy | 0 | 0 | 3 | 10 | gamma | 0 | O103:H- |
| EPEC52 | healthy | 1 | 0 | 3 |  | epsilon | 0 | O+:H45 |
| EPEC53 | healthy | 1 | 0 | 3 |  | alpha | 0 | O114:H- |
| EPEC54 | healthy | 0 | 1 | 1 | 21/29 | beta | 1 | O26:H- |
| EPEC55 | healthy | 0 | 0 | 3 | 28 | beta | 0 | O+:H6 |

**Suppl. Table 4.: LEE-subtypes and clonal groups of AEEC from different disease cohorts**

| **Cohort** | **Variables** | n, (%) | | | |
| --- | --- | --- | --- | --- | --- |
| **Crohn’s disease** | LEE1 | 1 | | (8%) |  |
|  | LEE2 | 0 | (0%) | |  |
|  | LEE3 | 11 | (92%) | |  |
|  | CG10 | 1 | (8%) | |  |
|  | CG21/29 | 1 | (8%) | |  |
|  | CG28 | 1 | (8%) | |  |
|  | CG526 | 3 | (25%) | |  |
|  | rare | 6 | (50%) | |  |
| **Ulcerative colitis** | LEE1 | 5 | | (38%) |  |
|  | LEE2 | 1 | (8%) | |  |
|  | LEE3 | 7 | (54%) | |  |
|  | CG3 | 1 | (8%) | |  |
|  | CG10 | 2 | (15%) | |  |
|  | CG21/29 | 1 | (8%) | |  |
|  | CG29 | 1 | (8%) | |  |
|  | rare | 8 | (62%) | |  |
| **Infectious diarrhea** | LEE1 | 2 | (17%) | |  |
|  | LEE2 | 3 | (25%) | |  |
|  | LEE3 | 7 | (58%) | |  |
|  | CG3 | 1 | (8%) | |  |
|  | CG10 | 1 | (8%) | |  |
|  | CG11 | 1 | (8%) | |  |
|  | CG28 | 2 | (17%) | |  |
|  | CG335 | 2 | (17%) | |  |
|  | CG517 | 1 | (8%) | |  |
|  | rare | 4 | (33%) | |  |
| **Healthy controls** | LEE1 | 3 | (15%) | |  |
|  | LEE2 | 0 | (0%) | |  |
|  | LEE3 | 7 | (85%) | |  |
|  | CG10 | 2 | (10%) | |  |
|  | CG28 | 4 | (20%) | |  |
|  | CG21/29 | 1 | (5%) | |  |
|  | CG29 | 1 | (5%) | |  |
|  | CG40 | 2 | (10%) | |  |
|  | CG526 | 2 | (10%) | |  |
|  | rare | 8 | (40%) | |  |

**Suppl. Table 5.: Primer and probes used for qPCR**

| Gene | Primer/Probe | Oligonucleotidesequence (5’🡪3’) | Size of Amplicon (bp) |
| --- | --- | --- | --- |
| uidA405^1^ | uidA405-F | CAACGAACTGAACTGGCAGA | 130 bp |
|  | uidA405-R | CATTACGCTGCGATGGAT |  |
|  | uidA405-P | CCCGCCGGGAATGGTGATTAC |  |
| eae^2^ | eae-F | CATTGATCAGGATTTTTCTGGTATA | 102 bp |
|  | eae-R | CTCATGCGGAAATAGCCGTTA |  |
|  | eae-P | ATAGTCTCGCCAGTATTCGCCACCAATACC |  |
| eaf^3^ | ep-f | GTTCTTGGCGAACAGGCTTGTC | 107 bp |
|  | ep-r | TTAAGCCAGCTACCATCCACC C |  |
|  | ep-p | AGTACTGACGTGCAGGTCGCCTGTTCG |  |
| aggR^4^ | aggR F | GAATCGTCAGCATCAGCTACA | 102 bp |
|  | aggR R | CCTAAAGGATGCCCTGATGA |  |
|  | aggR P | CGGACAACTGCAAGCATCTA |  |
| aaiC^4^ | aaiC F | CATTTCACGCTTTTTCAGGAAT | 160 bp |
|  | aaiC R | CCTGATTTAGTTGATTCCCTACG |  |
|  | aaiC P | CACATACAAGACCTTCTGGAGAA |  |
| Stx1^5^ | stx F | TTTGTYACTGTSACAGCWGAAGCYTTACG | 131 bp |
|  | stx R | CCCCAGTTCARWGTRAGRTCMACRTC |  |
|  | stx P | CTGGATGATCTCAGTGGGCGTTCTTATGTAA |  |
| stx2^5^ | stx2 F | TTTGTYACTGTSACAGCWGAAGCYTTACG | 128 bp |
|  | stx2 R | CCCCAGTTCARWGTRAGRTCMACRTC |  |
|  | stx P | TCGTCAGGCACTGTCTGAAACTGCTCC |  |
| sth^6^ | ST F | GCTAAACCAGYAGRGTCTTCAAAA | 147 bp |
|  | ST R | CCCGGTACARGCAGGATTACAACA |  |
|  | ST P | TGGTCCTGAAAGCATGAA |  |
| ipaH^7^ | ipaH F | CCT TTT CCG CGT TCC TTG A | 63 bp |
|  | ipaH R | CGG AAT CCG GAG GTA TTG C |  |
|  | ipaH P | CGC CTT TCC GAT ACC GTC TCT GCA |  |

1. Chern, E. C., Brenner, K. P., Wymer, L. & Haugland, R. A. Comparison of Fecal Indicator Bacteria Densities in Marine Recreational Waters by QPCR. *Water Qual. Expo. Heal.* **1**, 203–214 (2009).

2. Perelle, S., Dilasser, F., Grout, J. & Fach, P. Detection by 5′-nuclease PCR of Shiga-toxin producing Escherichia coli O26, O55, O91, O103, O111, O113, O145 and O157:H7, associated with the world’s most frequent clinical cases. *Mol. Cell. Probes* **18**, 185–192 (2004).

3. Hardegen, C. *et al.* A set of novel multiplex Taqman real-time PCRs for the detection of diarrhoeagenic Escherichia coli and its use in determining the prevalence of EPEC and EAEC in a university hospital. *Ann. Clin. Microbiol. Antimicrob.* **9**, 5 (2010).

4. Chaudhuri, R. R. *et al.* Complete genome sequence and comparative metabolic profiling of the prototypical enteroaggregative Escherichia coli strain 042. *PLoS One* **5**, (2010).

5. Nielsen, E. M. & Andersen, M. T. Detection and characterization of verocytotoxin-producing Escherichia coli by automated 5’ nuclease PCR assay. *J. Clin. Microbiol.* **41**, 2884–93 (2003).

6. Liu, J. *et al.* A Laboratory-Developed TaqMan Array Card for Simultaneous Detection of 19 Enteropathogens. **51**, 472–480 (2013).

7. Wang, S.-M. *et al.* Surveillance of shigellosis by real-time PCR suggests underestimation of shigellosis prevalence by culture-based methods in a population of rural China. *J. Infect.* **61**, 471–5 (2010).
