# Supplementary figures and images for: Atypical enteropathogenic E. coli are associated with disease activity in ulcerative colitis
Source: Gut Microbes. 2022 Nov 22;14(1):2143218. doi: 10.1080/19490976.2022.2143218 (PMC9704410; doi:10.1080/19490976.2022.2143218)

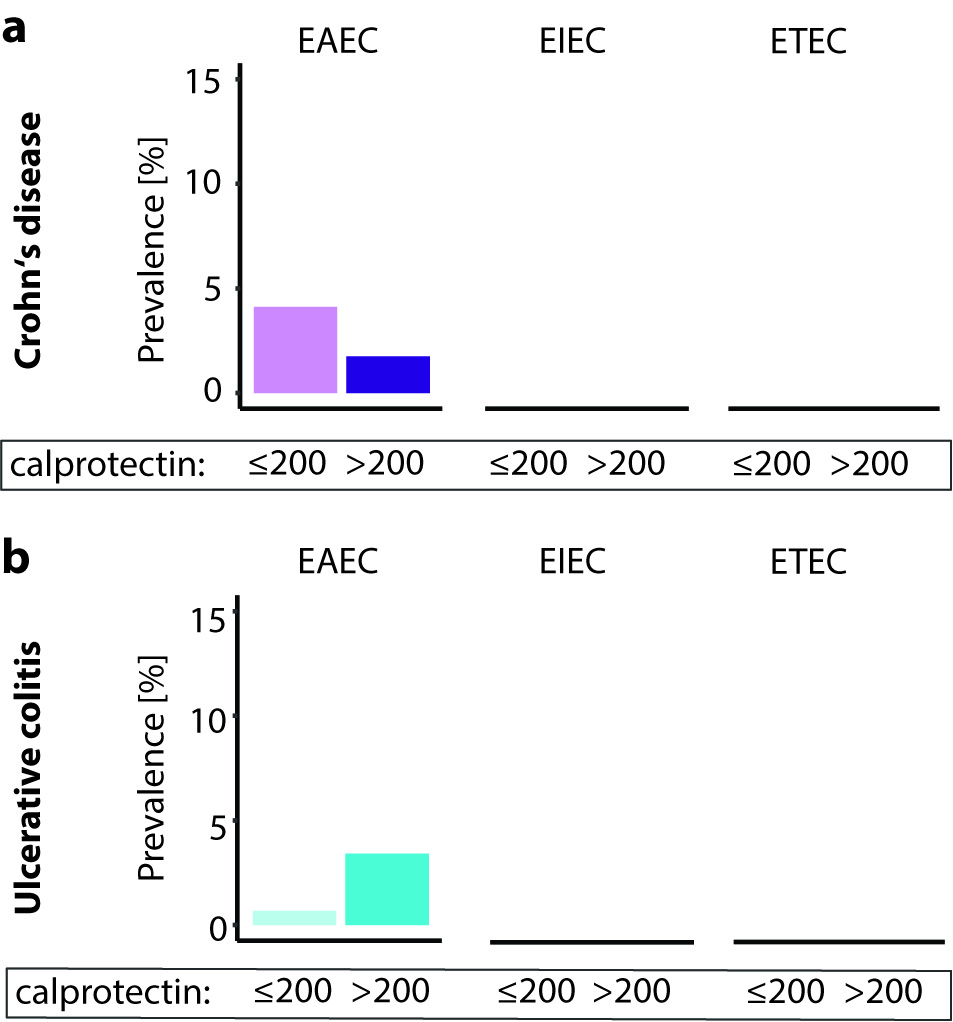

Supplement: Supplemental Material [file KGMI_A_2143218_SM2463.zip › FigS1.jpg]

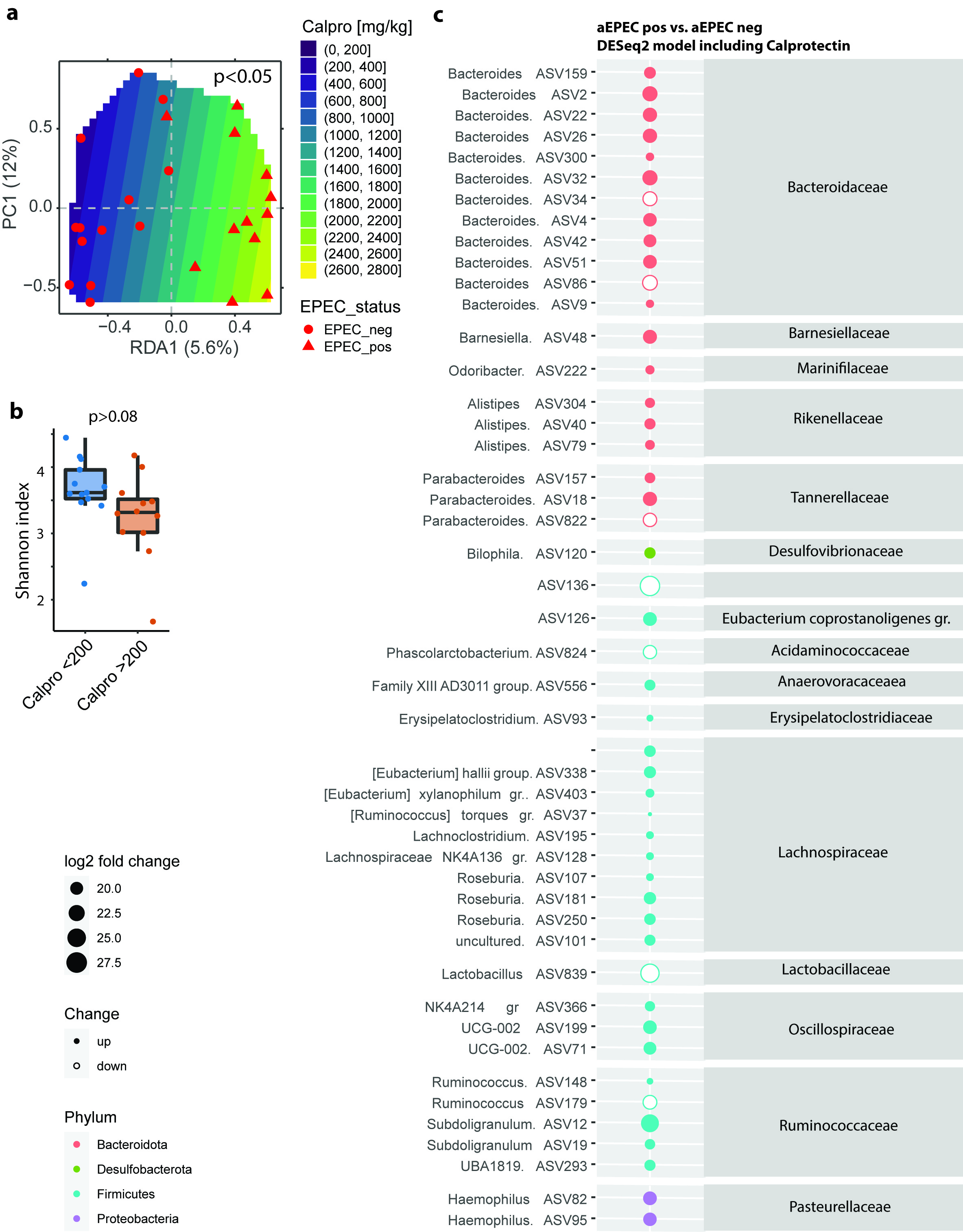

Supplement: Supplemental Material [file KGMI_A_2143218_SM2463.zip › FigS2.jpg]

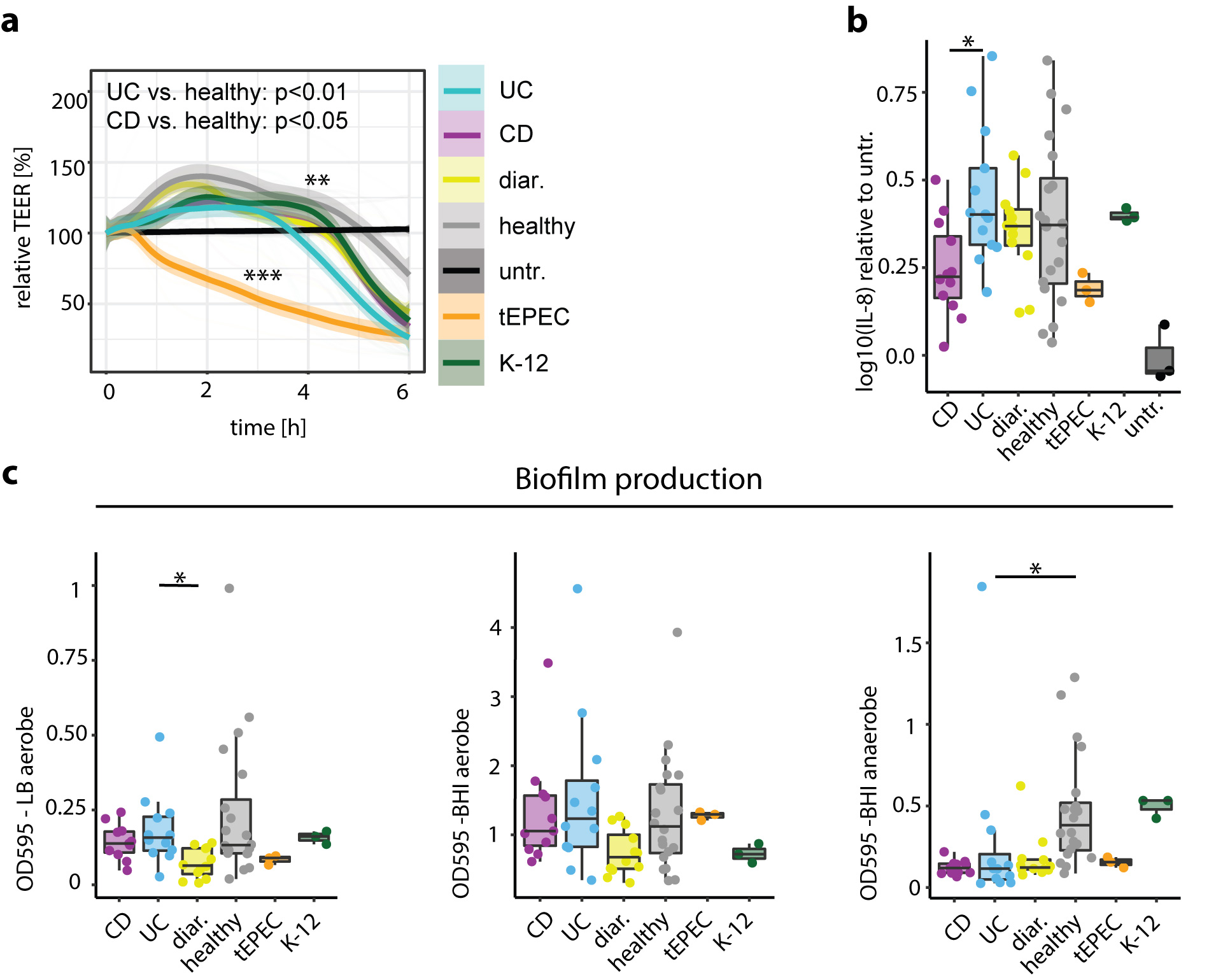

Supplement: Supplemental Material [file KGMI_A_2143218_SM2463.zip › FigS3.jpg]

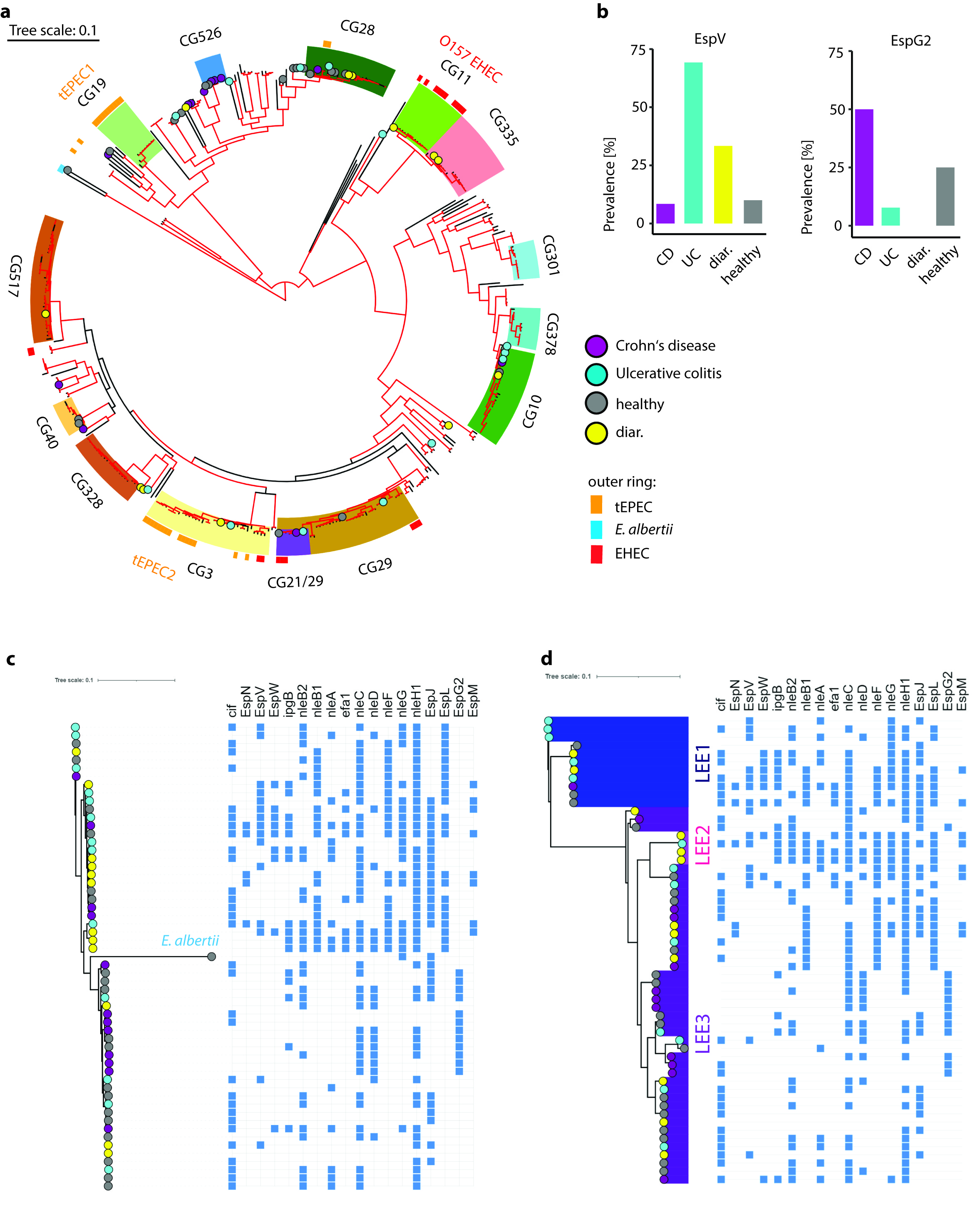

Supplement: Supplemental Material [file KGMI_A_2143218_SM2463.zip › FigS4.jpg]

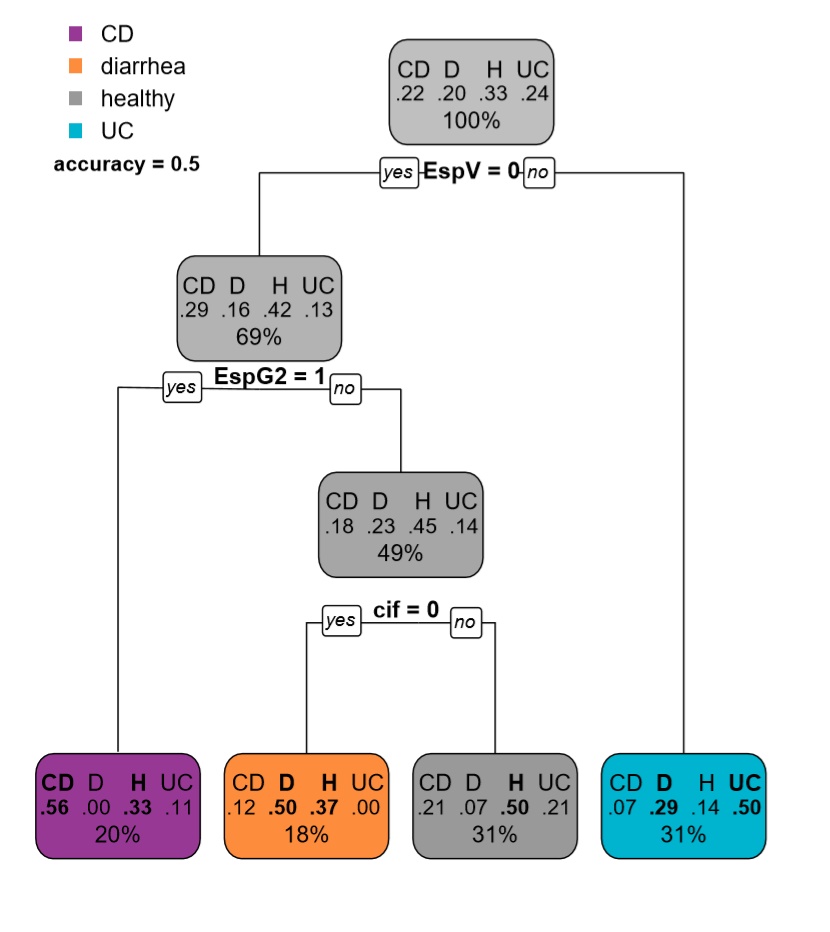

Supplement: Supplemental Material [file KGMI_A_2143218_SM2463.zip › FigS5.jpg]
